# Short and Long-Term Effects of the Angiotensin II Receptor Blocker Irbesartan on Intradialytic Central Hemodynamics: A Randomized Double-Blind Placebo-Controlled One-Year Intervention Trial (the SAFIR Study)

**DOI:** 10.1371/journal.pone.0126882

**Published:** 2015-06-01

**Authors:** Christian Daugaard Peters, Krista Dybtved Kjaergaard, Jens Dam Jensen, Kent Lodberg Christensen, Charlotte Strandhave, Ida Noerager Tietze, Marija Kristina Novosel, Bo Martin Bibby, Bente Jespersen

**Affiliations:** 1 Department of Renal Medicine, Aarhus University Hospital, Aarhus, Denmark; 2 Institute of Clinical Medicine, Aarhus University, Aarhus, Denmark; 3 Department of Cardiology, Aarhus University Hospital, Aarhus, Denmark; 4 Department of Nephrology, Aalborg University Hospital, Aalborg, Denmark; 5 Department of Medicine, Viborg Regional Hospital, Viborg, Denmark; 6 Department of Medicine, Fredericia Hospital, Fredericia, Denmark; 7 Department of Biostatistics, Aarhus University, Aarhus, Denmark; Kurume University School of Medicine, JAPAN

## Abstract

**Background and Aim:**

Little is known about the tolerability of antihypertensive drugs during hemodialysis treatment. The present study evaluated the use of the angiotensin II receptor blocker (ARB) irbesartan.

**Design:**

Randomized, double-blind, placebo-controlled, one-year intervention trial.

**Setting and Participants:**

Eighty-two hemodialysis patients with urine output >300 mL/day and dialysis vintage <1 year.

**Intervention:**

Irbesartan/placebo 300 mg/day for 12 months administered as add-on to antihypertensive treatment using a predialytic systolic blood pressure target of 140 mmHg in all patients.

**Outcomes and Measurements:**

Cardiac output, stroke volume, central blood volume, total peripheral resistance, mean arterial blood pressure, and frequency of intradialytic hypotension.

**Results:**

At baseline, the groups were similar regarding age, comorbidity, blood pressure, antihypertensive medication, ultrafiltration volume, and dialysis parameters. Over the one-year period, predialytic systolic blood pressure decreased significantly, but similarly in both groups. Mean start and mean end cardiac output, stroke volume, total peripheral resistance, heart rate, and mean arterial pressure were stable and similar in the two groups, whereas central blood volume increased slightly but similarly over time. The mean hemodynamic response observed during a dialysis session was a drop in cardiac output, in stroke volume, in mean arterial pressure, and in central blood volume, whereas heart rate increased. Total peripheral resistance did not change significantly. Overall, this pattern remained stable over time in both groups and was uninfluenced by ARB treatment. The total number of intradialytic hypotensive episodes was (placebo/ARB) 50/63 (*P* = 0.4). Ultrafiltration volume, left ventricular mass index, plasma albumin, and change in intradialytic total peripheral resistance were significantly associated with intradialytic hypotension in a multivariate logistic regression analysis based on baseline parameters.

**Conclusion:**

Use of the ARB irbesartan as an add-on to other antihypertensive therapy did not significantly affect intradialytic hemodynamics, neither in short nor long-term, and no significant increase in hypotensive episodes was seen.

**Trial registration:**

Clinicaltrials.gov NCT00791830

## Introduction

Hemodynamic instability is reported to occur in 4–30% of hemodialysis (HD) treatments [1–4]. It is not only highly unpleasant for the patients, but is also associated with higher patient morbidity and mortality [2, 5, 6]. Intravascular hypovolemia due to ultrafiltration (UF) is an essential factor, and pronounced intradialytic hypotension (IDH) rarely occurs with low UF volumes [7, 8].

The risk of instability may increase due to predisposing factors such as heart disease, diabetes, old age, atherosclerosis, food ingestion during dialysis, impaired sympathetic response, and antihypertensive medication [2, 9–11]. These factors should therefore also be considered in patients prone to IDH regardless of UF volume.

The renin-angiotensin-aldosterone system (RAAS) plays an important role in cardiovascular (CV) homeostasis due to its effects on vascular tone and volume. Short and long-term effects in end-stage renal disease patients attributed to RAAS include: vasoconstriction, sodium retention, cardiac hypertrophy, and arterial remodeling [12, 13]. RAAS-blockade with an angiotensin II receptor blocker (ARB) or an angiotensin-converting-enzyme inhibitor (ACEi) is known to improve CV outcome in patients without chronic renal failure [14, 15]. In HD patients, results have been contradictive and the value of RAAS-blockade has not been completely elucidated [16–20] although two recent meta-analyses reported a favorable effect of RAAS-blockade on left ventricular hypertrophy (LVH) [21, 22]. Regression of LVH is known to improve cardiac function, which in turn could lead to greater CV stability during HD and decreased risk of IDH. On the other hand, RAAS-blockade may impair the normal response to intravascular hypovolemia by blocking arteriolar constriction, and thus increase the risk of IDH. Furthermore, elevated potassium may discourage the use of RAAS-blockade in some HD patients [9, 23, 24].

To the best of our knowledge no previous studies have examined both acute and long-term effects of RAAS-blockade on intradialytic central hemodynamics in HD patients. Therefore, the aim of this study was to describe central hemodynamics during dialysis in detail in a group of newly started HD patients randomized to ARB (irbesartan) or placebo, aiming at a predialytic systolic blood pressure (BP) target of 140 mmHg in both groups. Beforehand, we hypothesized that ARB treatment would improve intradialytic hemodynamics.

## Methods

### Ethics

The study was conducted in accordance with good clinical practice (GCP) and the ethical standards described in the Helsinki Declaration. Written informed consent was obtained from all participants. The Central Denmark Region Committees on Biomedical Research Ethics, the Danish Health and Medicines Authority, and the Danish Data Protection Agency approved the study. Clinical Trials ID: NCT00791830.

### Study design

The study design and results on intermediate CV endpoints and residual renal function have previously been published [25–27]. Briefly, the SAFIR-study (acronym for”SAving residual renal Function in hemodialysis patients receiving IRbesartan) was designed as a double-blind multicenter randomized placebo-controlled trial primarily focusing on residual renal function and intermediate CV endpoints. The inclusion criteria were dialysis vintage <1 year, left ventricular ejection fraction >30% (echocardiography) and urine output >300 mL/day. Block randomization was applied to ensure equal distribution of patients with diabetes. Patients were recruited from six Danish hospitals and followed for one year. Inclusion began in May 2009. The last patient’s last visit took take place in December 2012. All sites were monitored by a local independent GCP-Unit.

### Study medication

The study medication consisted of the ARB irbesartan 150 mg, or matching placebo. The initial dose was one tablet per day. After two weeks, daily dose was increased to two tablets. Irbesartan is not removed by dialysis and timing of drug administration was not specified due to the long lasting effect of irbesartan (plasma half-life is 15 hours) [28, 29]. Compliance was checked monthly by counting residual tablets. Patients receiving RAAS-blocking agents at inclusion stopped this treatment one week before baseline. To reach equal BP-levels in the two treatment groups, investigators were instructed to achieve a predialytic systolic BP of 140 mmHg in all patients by adjusting dryweight and by use of all classes of antihypertensive drugs other than RAAS-blocking agents. Timing and intake of additional antihypertensive drugs was not specified.

### Blood pressure

BP was measured with validated automated oscillometric BP devices as previously described [25]. For assessment of the predialytic BP, a minimum of two measurements were performed and the average was used. Intradialytic BP was obtained at the same time as the intradialytic cardiac output (CO) measurements. Postdialytic BP was measured once in a non-standardized way (clinical routine) and was not used for adjustment of antihypertensive medication and dryweight assessment.

### Dialysis

Dialysate temperature and sodium concentrations followed routine prescriptions in the participating centers and were not standardized during the study period. Dialysate sodium concentrations were kept within the prescribed range by continuous online monitoring of the dialysate conductivity on the dialysis machines. UF volume was the actual volume removed during dialysis as registered on the dialysis machine. At baseline, 1 week, 3, 6, 9, and 12 months patients were not allowed to eat or drink within the first 30 minutes or the last 90 minutes of the dialysis session due to the intradialytic measurements, otherwise there we no special restrictions on fluid or food intake.

### Intradialytic measurements

Intradialytic hemodynamic parameters were evaluated at baseline, 1 week, 3, 6, 9, and 12 months, allowing for description of both short and long-term effects of ARB. CO was measured in duplicate by injecting a bolus of 30 mL 37^°^C isotonic saline into the venous blood line within the first and the last 30 minutes of the dialysis session using a previously validated method (Hemodialysis Monitor HD02/HD03, Flow-QC tubing sets, and clip-on flow/dilution sensors Transonic Systems Inc., Ithaca, NY,USA)[30–34]. Based on CO, the system estimates the volume of blood in the heart, lungs, and great vessels known as the central blood volume (CBV) [31]. The mean arterial blood pressure (MAP), total peripheral resistance (TPR), and stroke volume (SV) were derived by:

$$MAP=diastolic\;BP+\frac{\left(sytolic\;BP-diastolic\;BP\right)}{3}$$

$$CO=SV\;\times \;heart\;rate=\frac{MAP}{TPR}$$

Access recirculation (AR) can invalidate CO-measurements [31]. We used a built-in recirculation protocol to check for AR using injection of 10 mL isotonic saline into the venous blood line prior to the first CO-measurement. No measurements were performed if overt AR (pre-existing or induced by injection) was present. All injections were done within 4–7 seconds in order to minimize second pass, which can disrupt the dilution curve [31]. Loss of the saline indicator from the blood and into the tissues has previously been examined and is not considered a significant factor [35]. At end of study, seventy-five patients had at least one CO-measurement. Seven patients (placebo/ARB: 3/4) had no CO-measurements due to absence of an arteriovenous fistula (AV-fistula). Thus, prior to code breaking, 355 (out of 415 maximum theoretical) mean CO-values within the first 30 minutes of the HD-session were available for analysis. Sixty measurements were lacking due to absence of an AV-fistula (n = 52), equipment failure (n = 2) or because the measurements were not performed (n = 6). Additional twelve measurements within the last 30 minutes of the HD-session were missing due to technical problems. Hence, 343 mean CO-values within the last 30 minutes of the HD-session were available for analysis. A total of 144 access flow measurements were registered during the study period (placebo/ARB: 79/65) corresponding to at least one measurement in 21(49%)/23(56%) patients treated with placebo/ARB.

### Sample size and power calculations

As no data were available on central intradialytic hemodynamics in HD patients during the first year after commencing dialysis, power calculations were based on other endpoints (LVH, arterial stiffness and residual renal function) and showed a need for 22–24 patients in each group to complete the study. However, in expectation of 40% dropout (e.g. transplantation, adverse events), we decided to recruit 80 patients [25].

### Outcomes

The primary pre-specified outcome was intradialytic hemodynamic parameters: CO, MAP, SV, TPR, CBV, and heart rate (HR) [25]. Secondary outcome was the number of IDH episodes registered during the 12-month study period. All dialysis sessions in the study period were reviewed for IDH episodes defined as symptomatic hypotension requiring administration of intravenous fluid or preterm ending of the dialysis session.

### Statistics

Data were analyzed with Stata/IC 12.1 (StataCorp LP, College Station, TX 77845 USA). Intradialytic CV parameters (CO, SV, TPR, CBV, MAP, and HR) within the first and last 30 minutes of HD, changes in intradialytic CV parameters, pre-/postdialytic blood pressures, heart rate, UF volume, predialytic weight and plasma angiotensin II levels were analysed based on a multivariate repeated measurements model (xtmixed) with visit (baseline, 1 week, 3 months, 6 months, 9 months, and 12 months) and drug (placebo or ARB) and the interaction between them as factors allowing for missing values and dropout in the sense that it only excludes patients if all observations are missing for that patient. The mixed effects model is a population model in its nature so what we observe for those who complete the study will carry the most weight in the estimation of what happens in the full twelve-month period, but those who do not complete the study contribute with valuable information until they drop out. An approximate test for the hypothesis of equal standard deviations and correlations in the two groups was performed and the analysis was adjusted according to whether or not equal standard deviations and correlations were achieved. Model validation was performed by comparing observed and expected within subject standard deviations and correlations and by inspecting QQ-plots. Consequently, the two groups were compared regarding the development over time using four different models:

Model 1: Different development over time

Model 2: Parallel curves (same development over time)

Model 3: Equal levels in the two groups

Model 4: Constant curves (no change over time).

A likelihood ratio test (LR-test) was used to compare the models in order to describe the development over time. The first test compared Model 1 with Model 2. If the test was non-significant, we assumed parallel curves (same development over time). In case of parallel curves, we proceeded testing whether equal levels could be assumed by testing Model 2 versus Model 3. Finally, in case of equal levels in the two groups we tested whether there was a change over time (constant curves) by comparing Model 3 with Model 4. Pairwise comparisons between and within the placebo and ARB group were based on estimates from Model 1. Mean changes (baseline-12 months) and mean differences between groups were analyzed for selected parameters by using estimates from Model 1.

HD-time and antihypertensive medication could not be analyzed with this approach due to many identical values and Student’s t-test and/or Wilcoxon signed-rank test were used instead. Baseline data, adverse events, and distribution of antihypertensive drugs (all visits) were analyzed with chi-squared test (qualitative variables) and continuous variables were analyzed with t-test or Wilcoxon signed-rank test if normal distribution could not be assumed. Within group comparisons between start and end of dialysis at each visit was done with a paired t-test. Pearson's *r* was used to describe linear relationships. IDH episodes were dichotomized to 0 or ≥ 1 IDH events and used as outcome in univariate and multivariate logistic regression analysis based on various baseline parameters. Due to a relatively low number of IDH events only four parameters were used in the multivariate model. Three baseline parameters (UF volume, LV mass index, and low p-albumin (<3.9 g/dL)) were significant in a univariate analysis and were available in 81 out of 82 patients (LV data was missing in one patient due situs inversus (dextrocardia)). Therefore, the four-parameter multivariate model always included these three parameters. Different predictor variables were added and tested as the fourth variable. Intention-to-treat analyses were performed and *P*<0.05 was considered statistically significant. Values are presented as means with 95% confidence intervals unless otherwise stated.

## Results

### Patient characteristics and adverse events

**Fig 1:**
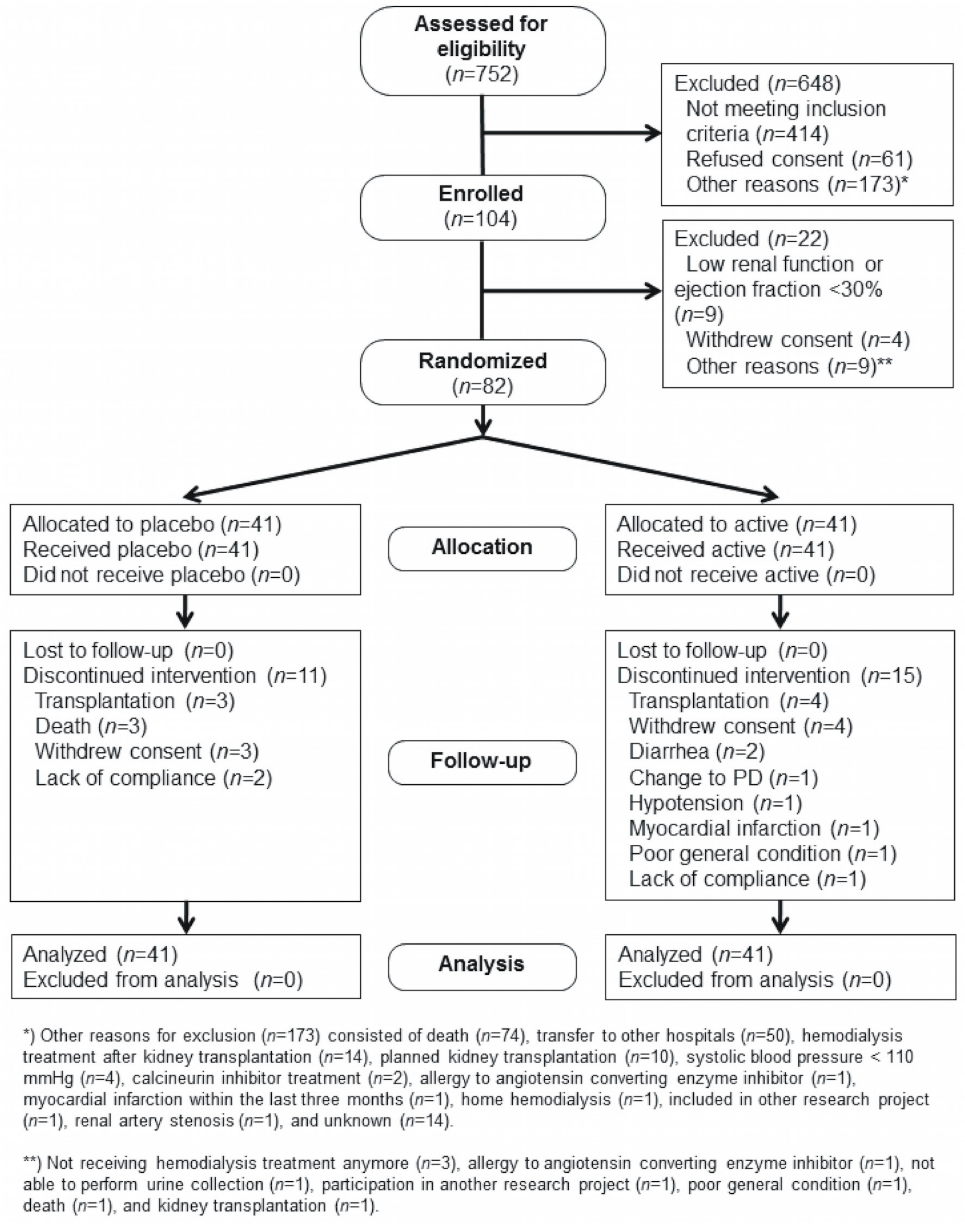
Consort flow diagram. This figure is reused from our previous publication [26]. Inclusion and exclusion criteria have been published previously [25]. Briefly, the main inclusion criteria were urine output >300 mL/day, dialysis vintage <1 year and left ventricular ejection fraction >30%. Absence of an AV-fistula was not an exclusion criterion since most patients were expected to obtain an AV-fistula during the study. Moreover, blood pressure and IDH episodes were monitored in all patients regardless of access modality. Time of dropout was not significantly different as previously reported [26].

Eighty-two patients were included in the study with forty-one in each group (Table 1). Overall, the groups were similar at baseline. Patients (placebo/ARB) were predominantly males (63/73%) with mean age 62/61 years, all had some residual urine production with a median output of (1.19/1.26 L/24 hours), and a relatively short median HD-vintage (137/148 days). Prevalences of CV disease (41/37%) and diabetes (29/32%) were similar. Twenty-six patients did not complete the study, eleven in the placebo and fifteen in the ARB group. Reasons for dropout are listed in Fig 1. Adverse events such as low/high BP or hyperkalemia were not significantly different in the two treatment groups as previously reported [26].

**Table 1:** Baseline characteristics.

		Placebo	ARB
Parameter	Unit	(*n* = 41)	(*n* = 41)
***Demographic characteristics***			
Age	years	62±14	61±16
Gender (males)	n (%)	26 (63)	30 (73)
Body weight	kg	81±17	79±17
Body mass index	kg/m^2^	28±5	26±5
Smokers	n (%)	11 (27)	14 (34)
Predialytic systolic BP	mmHg	145±19	148±21
Predialytic diastolic BP	mmHg	73±12	76±13
Predialytic heart rate	bpm	71±14	71±12
Diabetes	n (%)	12 (29)	13 (32)
Cardiovascular disease^‡^	n (%)	17 (41)	15 (37)
Ischemic heart disease	n (%)	9 (22)	8 (20)
LV mass index	g/m^2^	126±35	126±33
LV ejection fraction	%	60±9	62±9
Charlson comorbidity index^§^		3.5±1.6	3.8±1.9
***Primary renal disease***			
Diabetic nephropathy	n (%)	10 (24)	9 (22)
Glomerulonephritis	n (%)	4 (10)	6 (15)
Hypertensive renal disease	n (%)	7 (17)	6 (15)
Polycystic kidney disease	n (%)	6 (15)	2 (5)
Pyelonephritis	n (%)	2 (5)	2 (5)
Graft failure (previous Tx)#	n (%)	0 (0)	4 (10)
Other	n (%)	5 (12)	7 (17)
Unknown	n (%)	7 (17)	5 (12)
***Antihypertensive treatment***			
Pre-trial RAAS-blockade	n (%)	14 (34)	22 (54)
BP-drugs excl. Placebo/ARB	n	2.6±0.9	2.5±0.9
BP-drugs excl. Placebo/ARB	DDD	1.8±1.2	1.8±1.2
Beta-blocker	n (%)	27 (66)	27 (66)
Calcium channel blocker	n (%)	25 (61)	26 (63)
Alpha-blocker	n (%)	6 (15)	4 (10)
Comb. beta-alpha-blocker	n (%)	5 (12)	2 (5)
Other	n (%)	3 (7)	4 (10)
Loop diuretics	n (%)	38 (98)	40 (98)
Loop diuretics	DDD	6.3	12.5
***Other medication***			
Statin	n (%)	21 (51)	15 (37)
Aspirin	n (%)	21 (51)	16 (39)
Nitroglycerine	n (%)	6 (15)	4 (10)
Prednisolone	n (%)	1 (2)	5 (12)
EPO-treatment	n (%)	40 (98)	38 (93)
Alfacalcidol	n (%)	22 (54)	19 (46)
Calcium acetate	n (%)	18 (44)	20 (49)
Lanthanum	n (%)	4 (10)	2 (5)
Sevelamer	n (%)	9 (22)	9 (22)
***Dialysis parameters***			
Time on dialysis^c^	days	137 (53–431)	148 (54–400)
AV-fistula/central catheter	n (%)	36(88)/5(12)	32(78)/9(22)
Urine output^a^ ^,^ ^c^	L/24 hours	1.19 (0.16–2.72)	1.26 (0.27–3.19)
Glomerular filtration rate^b^	mL/min/1.73 m^2^	4.8±2.3	5.7±3.29
Modality (HDF/HD)	n (%)	3(7)/38(93)	1(2)/40(98)
Filter (low flux)	n (%)	22(54)	20(49)
Dialysate calcium conc.	mg/dL	5 (5–7)	5 (4–7)
Frequency	times/week	3 (2–3)	3 (2–4)
HD-time	hours/week	10±2	11±3
Ultrafiltration	L	1.3 (0.0–4.3)	0.6 (0.0–3.8)
Dry weight	kg	79±16	78±17
Urea reduction ratio	%	64±8	62±9
***Blood samples***			
Parathyroid hormone	pg/mL	117 (12–1286)	168 (16–516)
Albumin	g/dL	3.8±0.4	3.8±0.3
Hemoglobin	g/dL	10.9±1.5	11.1±1.3
Potassium	mEq/L	4.3±0.7	4.2±0.5

Data are presented as mean ± standard deviation or as median with range.

‡) Cardiovascular disease was defined as ≥ 1 known conditions (placebo/ARB): Ischemic heart disease (9/8); arrhythmia (6/5); valvular disease (6/3); heart failure (0/1); LV: Left ventricular;

#)Tx: Kidney transplantation;

§) Charlson comorbidity index score range 0–37; 0 = low, 3+ = high; DDD = Defined daily doses; EPO: erythropoetin;

a) n = 39 (Placebo group);

b) n = 36 (Placebo group);

GFR: glomerular filtration rate based on the mean of urinary creatinine and urea clearance as previously described [25]; bpm: beats per minute; HDF: hemodiafiltration; HD: hemodialysis;

c) Three patients in the placebo group and two patients in the ARB group had dialysis vintage > one year and four patients (two in each group) had urine output < 300 mL/day due to delay after inclusion/screening.

### Blood pressure and antihypertensive medication

**Fig 2:**
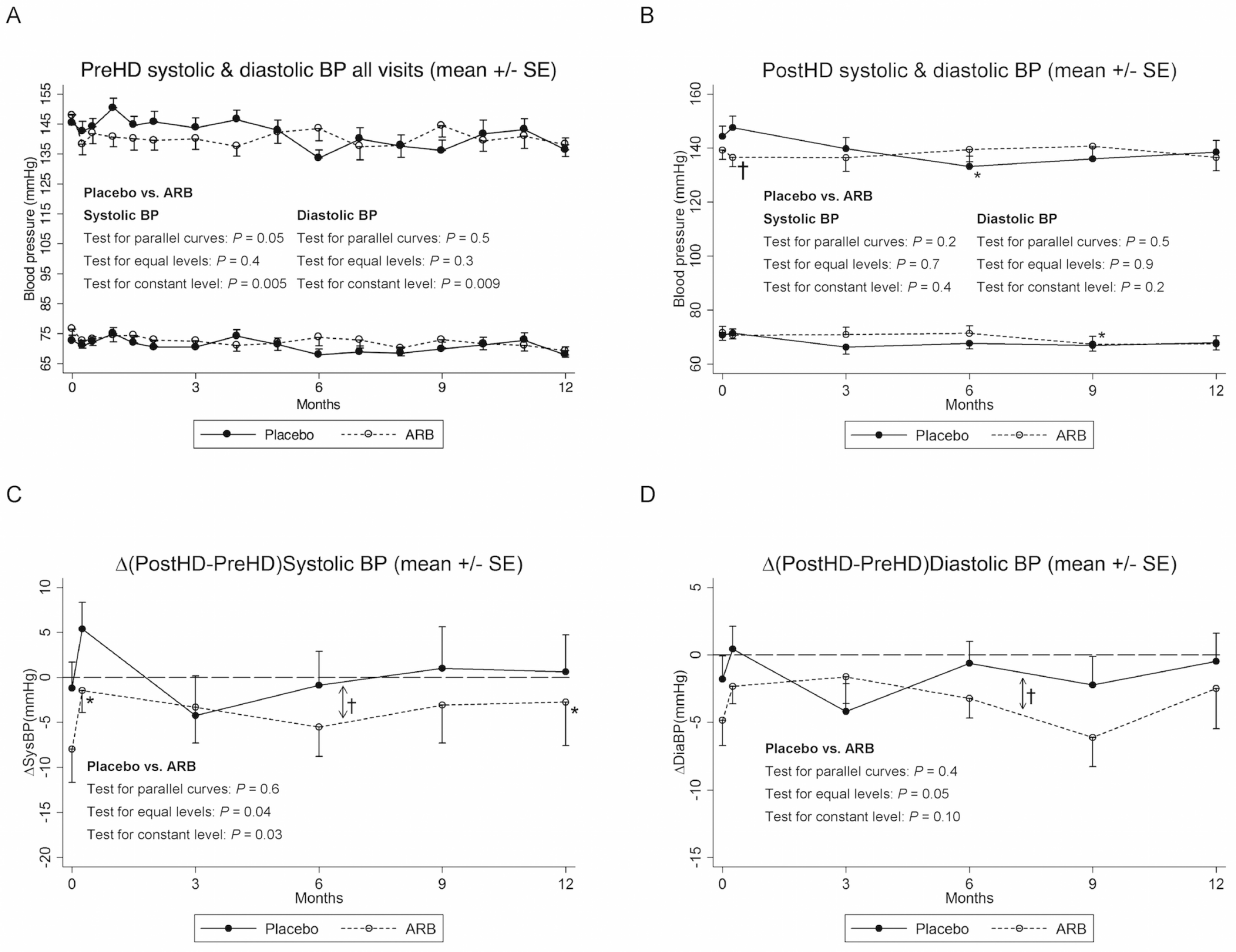
Pre- and postdialytic blood pressures. Fig 2A-B is reused from our previous publication but is slightly modified compared to the original. Image reproduced under a CC-BY licence from [26] with permission from Nature Publishing group. PreHD: Predialytic blood pressure (BP); PostHD: Postdialytic BP; Δ: PostHD-PreHD difference; *) 0.05>*P*≥0.01 vs. baseline within the placebo or ARB group; †) 0.05>*P* ≥0.01 placebo vs. ARB; ↕†) in Figs 2C-D indicates that parallel curves were assumed (Model 2). The constant mean differences (placebo-ARB) with 95% confidence interval were 6.2(0.5–11)mmHg; *P* = 0.03 (Δ(PostHD-PreHD)Systolic BP) and 3.0(0.1–6.0)mmHg; *P* = 0.04 (Δ(PostHD-PreHD)Diastolic BP). Note that Fig 2A shows all predialytic BP values recorded between baseline and 12 months, whereas the *P*-values given in 2A were based on values obtained at baseline, 1 week, 3 months, 6 months, 9 months, and 12 months, respectively from multivariate repeated meaurements analysis (xtmixed). Mean values from these time points are given in Table 2.

Predialytic systolic and diastolic BP were similar (tests for equal levels: *P>*0.3) and decreased significantly over time (tests for constant levels: *P*<0.01) as shown in Fig 2A (all recorded measurements) and Tables 2 and 3. Postdialytic systolic and diastolic BP (see Fig 2B) were also similar (test for parallel curves: *P*≥0.2; test for equal levels: *P*≥0.7) and constant over time (*P*≥0.2). S1 Table in the supplement summarizes all multivariate repeated measurements tests. Generally, the difference between pre- and postdialytic BP was small as shown in Fig 2C and 2D. During the study period this difference remained constant (tests for parallel curves: *P*≥0.4) indicating no significant effect of ARB. The constant mean differences were 6.2(0.5–11)mmHg (*P* = 0.03) (Δ(PostHD-PreHD)Systolic BP) and 3.0(0.1–6.0)mmHg (*P* = 0.04) (Δ(PostHD-PreHD)Diastolic BP). Use of additional antihypertensive medication in the study period besides placebo/ARB was similar in the two groups and there were no significant differences in defined daily doses, number or classes of additional BP-drugs. Plasma angiotensin II levels and tablet counts documented that patients were compliant as previously reported [26].

**Table 2:** Predialytic blood pressure, antihypertensive medication, and dialysis data.

			PreHD SysBP	PreHD DiaBP	PreHD heart rate	Additional BP-drugs	Additional BP-drugs	PreHD weight	HD-time	Ultra-filtration	Urine output	Access flow
Time	Group	n/n(AFM)	mmHg	mmHg	bpm	n	DDD	kg	h/w	L	L/24h	mL
**Baseline**	Placebo	41/12	145±19	73±12	71±14	2.6±0.9	1.8±1.2	80.9±17.3	9.8±2.3	1.32±1.27	1.30±0.71	1140±579
	ARB	41/13	148±21	76±13	71±12	2.5±0.9	1.8±1.2	78.7±17.0	10.7±3.1	1.20±1.36	1.45±0.79	1356±603
**1 week**	Placebo	39/12	143±21	71±11	72±13	2.6±1.0	1.8±1.3	81.9±17.5	9.8±2.2	1.28±1.26	1.28±0.71	1180±553
	ARB	40/12	138±22**	72±15**	70±11	2.6±0.9	1.9±1.2	79.3±17.2	10.5±2.4	1.19±1.30	1.33±0.75*	1395±612
**3 months**	Placebo	37/14	144±20	71±13	69±11	2.5±1.1	1.8±1.3	81.8±18.1	10.1±2.3	1.51±1.24	1.09±0.74*	996±497
	ARB	35/14	140±21**	72±12*	72±12	2.4±1.1	1.7±1.3	80.6±17.6**	10.5±2.6	1.28±1.25	1.36±0.78	1222±503
**6 months**	Placebo	33/13	134±16**	68±11*	73±10	2.3±1.1	1.4±1.2	80.5±19.9	10.5±2.2**	1.73±1.40	0.84±0.69***	1119±729
	ARB	30/8	143±22†	74±15	74±13	2.3±1.1	1.4±1.3*	79.8±17.5	10.5±2.6	1.00±1.15†	1.21±0.81†	1873±1000
**9 months**	Placebo	33/15	136±20**	70±13	74±12	2.3±1.2	1.5±1.3	81.0±19.1	10.9±2.1***	1.60±1.29	0.99±0.74***	1056±451
	ARB	29/10	144±20	73±15*	71±12	2.4±1.2	1.6±1.4	80.3±18.1*	11.3±4.4	1.56±1.42	1.21±0.82*	1233±567
**12 months**	Placebo	30/13	136±22*	68±15*	73±12	2.3±1.2	1.5±1.2	81.6±19.5	11.3±1.9**	1.74±1.29	0.93±0.88**	1109±447
	ARB	26/8	138±20*	69±11**	72±14	2.3±1.2	1.7±1.5	80.8±18.8	11.5±3.9	1.58±1.40	1.33±0.76†	1028±503

Values are given as means ± standard deviation.

*) 0.05>*P*≥0.01 vs. baseline within the placebo or ARB group;

**) 0.01>*P*≥0.001 vs. baseline within the placebo or ARB group;

***) *P*<0.001 vs. baseline within the placebo or ARB group;

†) 0.05>*P* ≥0.01 placebo vs. ARB; n(AFM): Number of patients with access flow measurement; DDD: Defined daily dose; SysBP: Systolic blood pressure; DiaBP; Diastolic blood pressure; bpm: beats per minute; h/w: hours per week. Although slightly modified this table is similar to Table 2 in our previous publication[26].

**Table 3:** Change (∆) between baseline and 12 months for selected parameters.

	ΔPlacebo		ΔARB		ΔPlacebo vs. ΔARB	
Parameter	Mean (95% CI)	*P*	Mean (95% CI)	*P*	Mean (95% CI)	*P*
***BP and HD data***						* *
PreHD SysBP (mmHg)	-8.2 (-16.2; -0.3)	**0.04**	-10.0 (-18.4; -1.7)	**0.02**	1.8 (-9.8; 13.3)	0.8
PreHD DiaBP (mmHg)	-4.0 (-7.9; -0.2)	**0.04**	-6.3 (-10.4; -2.3)	**0.002**	2.3 (-3.3; 7.9)	0.4
PostHD SysBP (mmHg)	-5.7 (-15.4; 4.0)	0.3	-5.1 (-15.3; 5.1)	0.3	-0.61 (-14.7; 13.5)	0.9
PostHD DiaBP (mmHg)	-1.7 (-6.4; 3.0)	0.5	-4.5 (-9.4; 0.5)	0.08	2.77 (-4.1; 9.6)	0.4
PreHD weight (kg)	-1.9 (-3.9; 0.2)	0.08	1.3 (-0.5; 3.1)	0.2	-3.2 (-5.9; -0.4)	**0.03**
Ultrafiltration (mL)	263 (-103; 629)	0.2	281 (-81; 643)	0.1	-17 (-532; 497)	0.9
Urine output (mL/24h)	-453 (-697; -208)	**<0.001**	-240 (-464; -16)	**0.04**	-213 (-545; 119)	0.2
HD time (hours/week)^a^	1.4 (0.6; 2.1)	**<0.001**	0.5 (-1.1; 2.1)	0.6	0.9 (-0.9; 2.6)	0.3
Additional BP-drugs (DDD) ^a^	-0.1 (-0.4; 0.2)	0.6	-0.3 (-0.8; 0.2)	0.2	0.2 (-0.3; 0.8)	0.5
***Blood samples***						
Hemoglobin (g/dL)	0.5 (0.01; 1.08)	**0.04**	0.1 (-0.45; 0.7)	0.7	0.4 (-0.3; 1.2)	0.3
log(Ang2) (log(pg/mL))	-0.2 (-0.6; 0.1)	0.2	0.4 (0.1; 0.8)	**0.01**	-0.7 (-1.1; -0.2)	**0.01**
***HD***_***START***_						
CO1 (L/min)	-0.1 (-0.5; 0.4)	0.8	-0.2 (-0.7; 0.4)	0.6	0.1 (-0.6; 0.8)	0.8
MAP1 (mmHg)	-3.3 (-8.8; 2.2)	0.2	-5.1 (-11.2; 0.9)	0.1	1.8 (-6.4; 10.0)	0.7
HR1 (bpm)	4.1 (-1.5; 9.6)	0.2	0.6 (-4.5; 5.7)	0.8	3.5 (-4.1; 11.0)	0.4
TPR1 (mmHg/(L/min))	-0.6 (-2.1; 0.9)	0.4	-0.3 (-2.0; 1.3)	0.7	-0.3 (-2.5; 2.0)	0.8
CBV1 (L)	0.1 (0.0; 0.2)	**0.03**	0.1 (-0.1; 0.2)	0.5	0.0 (-0.1; 0.2)	0.6
SV1 (mL)	-4.9 (-14.7; 5.0)	0.3	-5.6 (-15.1; 3.9)	0.3	0.7 (-12.9; 14.4)	0.9
***HD***_***END***_						
CO2 (L/min)	0.0 (-0.6; 0.6)	0.9	-0.1 (-0.8; 0.7)	0.9	0.1 (-0.9; 1.0)	0.9
MAP2 (mmHg)	-1.7 (-7.0; 3.6)	0.5	-3.6 (-9.5; 2.2)	0.2	1.9 (-6.0; 9.8)	0.6
HR2 (bpm)	2.4 (-1.8; 6.5)	0.3	1.6 (-4.6; 7.8)	0.6	0.8 (-6.7; 8.2)	0.8
TPR2 (mmHg/(L/min))	0.0 (-1.7; 1.6)	0.9	-1.4 (-2.7; -0.2)	**0.02**	1.4 (-0.7; 3.5)	0.2
CBV2 (L)	0.1 (0.0; 0.3)	0.1	0.1 (0.0; 0.2)	**0.03**	0.0 (-0.2; 0.2)	0.9
SV2 (mL)	-3.0 (-11.8; 5.8)	0.5	-1.9 (-12.7; 9.0)	0.7	-1.1 (-15.1; 12.8)	0.9
***Δ(HD***_***END***_ ***-HD***_***START***_ ***)***						
ΔCO (L/min)	-0.1 (-0.9; 0.6)	0.7	0.4 (-0.1; 1.0)	0.2	-0.5 (-1.5; 0.4;)	0.3
ΔMAP (mmHg)	1.1 (-4.0; 6.3)	0.7	1.2 (-3.7; 6.2)	0.6	-0.1 (-7.2; 7.0)	0.9
ΔHR (bpm)	-0.8 (-4.3; 6.0)	0.8	1.0 (-4.5; 6.5)	0.7	-1.8 (-9.3; 5.7)	0.6
ΔTPR (mmHg/(L/min))	0.9 (-1.0; 2.8)	0.4	-0.9 (-2.2; 0.3)	0.1	1.8 (-0.5; 4.1)	0.1
ΔCBV (L)	-0.0 (-0.1; 0.1)	0.9	0.1 (-0.0; 0.2)	0.1	-0.1 (-0.2; 0.1)	0.4
ΔSV (mL)	-1.9 (-11.8; 8.0)	0.7	5.6 (-3.9; 15.1)	0.3	-7.5 (-21.2; 6.2)	0.3

Change baseline-12 months (Δ) for selected parameters. Values are given as means with 95% confidence intervals (95% CI). Estimates and *P*-values were based on multivariate repeated measurements model (xtmixed) Model 1 assuming different development over time in the two groups (placebo and ARB).

a) HD-time and additional antihypertensive drugs besides placebo/ARB could not be analyzed with xtmixed due to many identical values. Differences within/between groups were therefore analyzed with paired/unpaired Student’s t-test. HD_START_: Measurements performed within the first 30 minutes of the HD-session; HD_END_: Measurements performed within the last 30 minutes of the HD-session; DDD: Defined daily doses; Ang2: Angiotensin II Hemoglobin conversion factor from g/dL to mmol/L is x 0.6206.

### Dialysis parameters

Access modality at baseline is shown in Table 1. After 12 months, 97% had an AV-fistula in the placebo group compared to 92% in the ARB group (*P* = 0.5). Mean access flow and HD-time did not differ significantly between groups (Tables 2 and 3). Overall, UF volume and urine output were not significantly different in the two groups (test for equal levels: *P* = 0.3 (UF volume); *P* = 0.3 (urine output)). In both groups, UF volume tended to increase over time (test for constant level: *P* = 0.06), whereas urine output decreased significantly over time (test for constant level: *P*<0.001). Predialytic weight tended to decrease between baseline and 12 months in the placebo group, whereas in the ARB group it tended to increase. Mean difference (∆Placebo PreHD weight over one year—∆ARB PreHD weight over one year) was -3.2(-0.4; -5.9)kg (*P* = 0.03) as shown in Table 3.

### Intradialytic hemodynamic parameters: Within-group comparisons

Intradialytic hemodynamic parameters obtained within the first (HD_START_) and last (HD_END_) 30 minutes of the dialysis session in the two groups are summarized in Table 4. During a HD-session, CO, MAP, CBV, and SV decreased whereas HR increased from HD_START_ to HD_END_ although not always significantly_._ TPR did not change significantly.

**Table 4:** Intradialytic hemodynamic parameters within group comparisons of HD_START_ vs. HD_END_.

			Cardiac output		Mean blood pressure		Heart rate		Total peripheral resistance		Stroke volume		Central blood volume	
			L/min		mmHg		bpm		mmHg*min/L		mL		L	
Time	Group	n_START_/n_END_	HD_START_	HD_END_	HD_START_	HD_END_	HD_START_	HD_END_	HD_START_	HD_END_	HD_START_	HD_END_	HD_START_	HD_END_
**Baseline**	Placebo	35/33	6.2±1.6	5.8±1.6*	96±12	91±15*	69±12	72±12	16.3±3.7	16.8±4.1	92±26	79±20**	1.0±0.3	1.0±0.3
	ARB	31/30	6.8±1.9	6.3±1.5*	98±13	92±17	69±12	74±16**	15.6±4.6	15.5±4.7	101±32	89±26**	1.1±0.4	1.0±0.3**
**1 week**	Placebo	33/31	6.3±1.7	6.0±1.5	95±14	92±17*	70±12	73±11*	15.7±3.1	15.8±3.5	92±26	85±29*	1.0±0.3	1.0±0.3*
	ARB	31/30	6.8±2.1	6.3±1.9*	96±15	89±14**	70±12	73±15**	15.1±4.5	15.3±4.2	103±34	89±28**	1.2±0.4	1.0±0.3***
**3 months**	Placebo	33/32	6.1±1.9	5.6±1.5**	94±13	88±13*	68±11	71±11**	16.3±4.1	16.8±3.9	90±22	78±19***	1.0±0.3	1.0±0.3*
	ARB	30/28	6.5±1.7	5.9±1.5*	93±12	86±14*	68±11	73±16**	15.3±4.8	15.5±4.5	99±32	84±26***	1.1±0.4	1.1±0.3*
**6 months**	Placebo	30/28	6.0±2.4	5.6±1.8	87±13	89±11	69±10	72±10*	16.1±4.7	17.6±5.8*	86±27	78±24*	1.0±0.5	1.0±0.3
	ARB	25/25	6.4±1.7	6.0±1.1	94±16	88±13**	69±10	75±15*	15.7±5.3	15.1±3.5	94±28	83±19**	1.1±0.4	1.1±0.4
**9 months**	Placebo	28/28	6.2±1.7	5.7±1.6*	90±12	88±15	72±12	76±10*	15.4±3.4	16.2±4.1	87±22	77±23***	1.1±0.3	1.0±0.3**
	ARB	26/25	6.4±1.8	6.1±1.8	96±18	87±13*	72±12	76±16**	16.0±4.9	15.1±4.0	92±24	82±25**	1.2±0.4	1.1±0.3*
**12 months**	Placebo	29/29	6.0±1.5	5.5±1.7	91±16	87±17	73±14	74±13	15.8±4.2	16.9±4.6	86±22	76±24*	1.1±0.3	1.1±0.5
	ARB	24/24	6.2±1.6	6.2±1.4	92±10	88±13	73±14	74±15*	15.9±4.3	15.0±3.8	91±21	86±23	1.1±0.4	1.1±0.4

Values are given as mean ± standard deviation

*) 0.05>*P*≥0.01 vs. HD_START_ within the placebo or ARB group;

**) 0.01>*P*≥0.001 vs. HD_START_ within the placebo or ARB group;

***) *P*<0.001 vs. HD_START_ within the placebo or ARB group; HD_START_: Measurements performed within the first 30 minutes of the HD-session; HD_END_: Measurements performed within the last 30 minutes of the HD-session; bpm: Beats per minute

### Between-group comparisons and development over time

Regardless of the time of assessment, there was no significant difference in CO, MAP, HR, TPR, and SV between placebo and ARB over time (S1 and S2 Figs) and these parameters were stable during the study period (*P*≥0.07 in all tests for parallel curves, equal levels, and constant levels). CBV increased significantly but similarly in the two groups (test for equal levels: *P* = 0.07 (HD_START_); *P* = 0.3 (HD_END_)) during the study period both when measured at HD_START_ (test for constant level: *P* = 0.008) and when measured at HD_END_ (test for constant level: *P* = 0.005).

### Intradialytic changes (HD_END_-HD_START_) between-group comparisons and development over time

**Fig 3:**
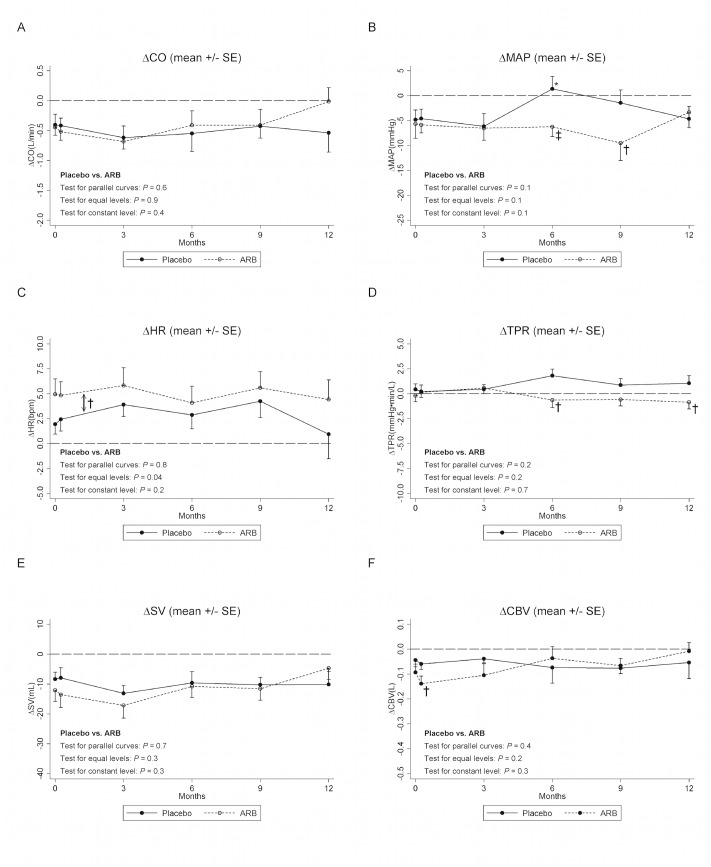
Intradialytic hemodynamic changes. Δ = (HD_END_—HD_START_); HD_START_: Intradialytic measurement within the first 30 minutes of dialysis; HD_END_: Intradialytic measurement within the last 30 minutes of dialysis; CO: Cardiac output; MAP: Mean arterial blood pressure; HR: Heart rate; TPR: Total peripheral resistance; SV: Stroke volume; CBV: Central blood volume (volume of blood in the heart, lungs, and the great vessels). *) 0.05>*P*>0.01 vs. baseline within the placebo or ARB group †) 0.05>*P*>0.01 vs. placebo ‡) 0.01>*P* >0.001 vs. placebo ↕†) Indicates that parallel curves were assumed (Model 2). The constant mean difference (placebo-ARB) with 95% confidence interval was 2.4(0.2.4.6) bpm (*P* = 0.03).

Fig 3 shows intradialytic changes (Δ = HD_END_-HD_START_). Overall, there was no significant difference in ΔCO, ΔMAP, ΔTPR, ΔSV, and ΔCBV between placebo and ARB (*P*≥0.1 in all tests for parallel curves and equal levels). Thus, taking all twelve months into account we found no significant difference in ΔMAP between placebo and ARB (test for parallel curves: *P* = 0.1; test for equal levels: *P* = 0.1) although ΔMAP was significantly lower in the ARB group at six and nine months. Mean difference in ΔMAP was 9(3–15) and 8(0–16) mmHg (*P* = 0.006; *P* = 0.04) at six and nine months, respectively. Similarly, taking all twelve months into account we found no significant difference in ΔTPR (test for parallel curves: *P* = 0.2; test for equal levels: *P* = 0.2) although ΔTPR was significantly lower in the ARB group at six and twelve months. Mean difference in ΔTPR was 2.0(0.2–3.8) and 2.1(0.4–3.9) mmHg min/L (*P* = 0.03; *P* = 0.02) at six and twelve months, respectively. ΔHR levels differed significantly between groups at baseline with a slightly higher mean intradialytic increase in HR in the ARB group corresponding to a mean ΔHR difference of 2.4(0.2–4.6) bpm (*P* = 0.03). During the study period the difference remained roughly constant (test for parallel curves: *P* = 0.2) indicating no significant effect of ARB. Additional secondary findings are presented in the supplement (S1 Text).

### Intradialytic hypotension

IDH occurred in less than 1% of all dialysis treatments in the study period, much less than expected, and the total number of IDH episodes (placebo/ARB) was 50/63 (*P* = 0.4). The groups were similar in terms of the number of patients without IDH episodes (placebo/ARB) 28/23 and patients with one or more IDH episodes 13/18 (*P* = 0.3). In univariate logistic regression analysis based on baseline parameters, UF volume, LV mass index, p-albumin <3.9 g/dL were significantly associated with IDH as shown in Table 5. Glomerular filtration rate, urine output, and intradialytic MAP and SV obtained within the last 30 minutes of the dialysis session were also significantly associated with IDH, but neither of them were significant in multivariate analysis as shown in Table 5. Thus, in multivariate logistic regression analysis based on baseline parameters we found that UF volume, LV mass, p-albumin<3.9 g/dL, and ΔTPR were significantly associated with IDH (Table 5). Adjusted odds ratios (OR) were: 1.99(1.28; 3.07) (UF volume); 0.98(0.96; 0.99) (LV mass index); 3.76(1.31; 10.80) (p-albumin<3.9 g/dL), and 0.77(0.61; 0.97) (ΔTPR). The odds for an IDH event were not significantly increased on ARB treatment compared to placebo neither in univariate analysis (OR: 1.69(0.68; 4.15), *P* = 0.3), nor when adjusted for baseline UF volume, LV mass, and plasma albumin level (OR: 1.70(0.59; 4.89), *P* = 0.3). Although time of dropout was not significantly different in the two groups as previously reported [26], additional adjustment for time in the study resulted in reduced odds for an IDH event on ARB treatment (OR 1.61(0.55; 4.72), *P* = 0.4).

**Table 5:** Correlates of intradialytic hypotension by univariate and multivariate logistic regression.

		Univariate analysis			Multivariate analysis		
Parameter		OR	95% CI	*P*	OR	95% CI	*P*
***BP and BP-lowering drugs***		** **	** **	*** ***	** **	** **	*** ***
Predialytic systolic BP (mmHg)		1.00	(0.98; 1.02)	0.9	1.01	(0.98; 1.03)	0.5
ARB treatment		1.69	(0.68; 4.15)	0.3	1.70	(0.59; 4.89)	0.3
Antihypertensive drugs (number of drugs)		1.13	(0.69; 1.85)	0.6	1.10	(0.62; 1.96)	0.8
Antihypertensive drugs (defined daily doses)		0.99	(0.68; 1.43)	0.9	1.18	(0.73; 1.90)	0.5
***Demographic parameters***							*** ***
Time in study (days)		1.00	(1.00; 1.01)	0.2	1.00	(0.99; 1.00)	0.5
Age (years)		1.00	(0.97; 1.03)	0.8	1.02	(0.98; 1.06)	0.4
Female gender		1.04	(0.40; 2.71)	0.9	1.10	(0.33; 3.68)	0.9
Body mass index (kg/m^2^)		1.01	(0.92; 1.10)	0.9	0.94	(0.84; 1.05)	0.3
Deviation from dryweight (weight-dryweight)		1.22	(0.96; 1.56)	0.1	0.94	(0.60; 1.48)	0.8
Diabetes		1.45	(0.56; 3.79)	0.4	0.81	(0.26; 2.50)	0.7
Heart disease		1.22	(0.49; 3.03)	0.7	1.30	(0.45; 3.78)	0.6
Charlson comorbidity index ≥5		1.79	(0.67; 4.76)	0.2	1.54	(0.49; 4.91)	0.5
PWV_SD (m/s)		1.04	(0.89; 1.21)	0.6	1.00	(0.84; 1.19)	0.9
LV mass index (g/m^2^)		0.98	(0.97; 1.00)	**0.05**	0.98	(0.96; 0.99)	**0.009**
GFR (mL/min/1.73m^2^)		0.84	(0.70; 1.00)	**0.05**	0.92	(0.74; 1.14)	0.5
Urine output (mL/kg/day)		0.94	(0.89; 0.99)	**0.01**	0.97	(0.91; 1.04)	0.4
***Blood samples***							
Low albumin (<3.9 g/dL)		3.33	(1.31; 8.48)	**0.01**	3.76	(1.31; 10.80)	**0.01**
Sodium (mmol/L)		0.87	(0.75; 1.01)	0.1	0.93	(0.77; 1.12)	0.5
Hemoglobin (g/dL)		0.88	(0.54; 1.45)	0.6	0.90	(0.62; 1.32)	0.6
Adrenaline (nmol/L)		0.00	(0.00; 2.41)	0.1	0.00	(0.00; 2.77)	0.1
Noradrenaline (nmol/L)		0.74	(0.52; 1.05)	0.1	0.94	(0.60; 1.45)	0.8
log(NT-proBNP (pg/mL))		1.06	(0.73; 1.53)	0.8	1.02	(0.63; 1.68)	0.9
log(CRP (μg/mL))		1.28	(0.90; 1.83)	0.2	1.26	(0.83; 1.92)	0.3
***Heart rate variability***							
SDNN (ms)		0.98	(0.95; 1.02)	0.3	0.98	(0.94; 1.02)	0.2
LF/HF ratio		0.89	(0.63; 1.25)	0.5	1.06	(0.70; 1.62)	0.8
Valsalva ratio		11.28	(0.12; 1047.89)	0.3	154.92	(0.63; 38301.38)	0.1
Stand ratio		0.81	(0.02; 26.84)	0.9	0.65	(0.02; 24.87)	0.8
***Dialysis related parameters***							
HD-vintage (days)		1.00	(0.99; 1.00)	0.3	1.00	(0.99; 1.00)	0.2
HD frequency	x2	0.44	(0.15; 1.29)	0.1	0.84	(0.23; 3.10)	0.8
	x4	1.33	(0.08; 22.41)	0.8	0.97	(0.04; 26.00)	1
Weekly HD time	<8 hours	0.50	(0.15; 1.70)	0.3	0.85	(0.21; 3.54)	0.8
	>12 hours	1.25	(0.44; 3.58)	0.7	1.05	(0.29; 3.80)	0.9
Ultrafiltration volume (liter)		1.58	(1.11; 2.26)	**0.01**	1.99	(1.28; 3.07)	**0.002**
High flux HD filter		0.98	(0.40; 2.38)	0.9	1.17	(0.41; 3.35)	0.8
Dialysate calcium>1.25 mmol/L		1.36	(0.45; 4.12)	0.6	1.77	(0.50; 6.34)	0.4
Access modality dialysis catheter		0.38	(0.12; 1.24)	0.1	0.41	(0.11; 1.59)	0.2
***Hemodynamic parameters***							
**HD**_**START**_	CO (L/min)	0.93	(0.70; 1.24)	0.6	0.81	(0.56; 1.17)	0.3
	MAP (mmHg)	0.99	(0.95; 1.03)	0.6	1.01	(0.96; 1.06)	0.7
	HR (bpm)	1.02	(0.98; 1.07)	0.2	1.03	(0.98; 1.08)	0.2
	TPR (mmHg/(L/min))	1.03	(0.91; 1.16)	0.7	1.11	(0.96; 1.30)	0.2
	CBV (L)	1.30	(0.32; 5.24)	0.7	0.68	(0.12; 3.81)	0.7
	SV (mL)	0.99	(0.97; 1.01)	0.3	0.98	(0.95; 1.00)	0.1
**HD**_**END**_	CO (L/min)	0.84	(0.59; 1.18)	0.3	0.91	(0.61; 1.34)	0.6
	MAP (mmHg)	0.96	(0.92; 1.00)	**0.05**	0.98	(0.94; 1.02)	0.2
	HR (bpm)	1.04	(1.00; 1.08)	0.1	1.04	(0.99; 1.09)	0.1
	TPR (mmHg/(L/min))	0.97	(0.86; 1.10)	0.7	0.98	(0.87; 1.12)	0.8
	CBV (L)	0.90	(0.16; 5.03)	0.9	0.82	(0.09; 7.29)	0.9
	SV (mL)	0.97	(0.95; 1.00)	**0.05**	0.98	(0.95; 1.01)	0.1
**Δ(HD**_**END**_ **-HD**_**START**_ **)**	ΔCO (L/min)	0.95	(0.59; 1.53)	0.8	1.84	(0.88; 3.87)	0.1
	ΔMAP (mmHg)	0.96	(0.92; 1.01)	0.1	0.96	(0.92; 1.10)	0.1
	ΔHR (bpm)	1.08	(1.00; 1.17)	0.1	1.07	(0.97; 1.17)	0.2
	ΔTPR (mmHg/(L/min))	0.90	(0.75; 1.07)	0.2	0.77	(0.61; 0.97)	**0.02**
	ΔCBV (L)	0.30	(0.01; 6.46)	0.4	3.91	(0.11; 145.52)	0.5
	ΔSV (mL)	0.99	(0.96; 1.02)	0.4	1.02	(0.97; 1.06)	0.5

The table shows odds ratios from univariate and multivariate logistic regression analysis based on baseline data with intradialytic hypotension (IDH) as outcome with adjustment for random patient effect (for 1 unit change in the predictor variable) or comparison between one or more groups (e.g. placebo vs. ARB treatment). The model always included ultrafiltration volume, LV mass index, and plasma albumin. Different predictor variables were added and tested as the fourth variable. IDH episodes were dichotomized to 0 or ≥1 IDH events. HD frequency, HD filter, weekly HD time and access modality references were x3 per week, low flux filter, 8–12 hours/week, and AV-fistula, respectively. Plasma albumin concentration, Charlson comorbidity index, and dialysate calcium concentration were dichotomized with ≥3.9 g/dL (albumin), <5 (Charlson), and 1.25 mmol/L (dialysate calcium) as references, respectively. 95% CI: 95% confidence interval; CRP: Plasma C-reactive protein; NT-proBNP: N-terminal prohormone of brain natriuretic peptide; GFR: Glomerular filtration rate; BP: Blood pressure; LV mass index: Left ventricular mass index; PWV_SD: Pulse wave velocity (subtracted distance) obtained as previously described[26]; SDNN: The standard deviation of the N-N interval obtained by analysis of heart rate variability (HRV) see reference [26] for details; LF/HF ratio: Ratio between low frequency (LF) power and high frequency (HF) power from HRV analysis. HD: Hemodialysis. HD_START_: Measurements performed within the first 30 minutes of the HD-session; HD_END_: Measurements performed within the last 30 minutes of the HD-session; CO: Cardiac output; MAP: Mean arterial blood pressure; HR: Heart rate; bpm: beats per minute; TPR: Total peripheral resistance; SV: Stroke volume; CBV: Central blood volume (volume of blood in the heart, lungs, and the great vessels)

## Discussion

To our knowledge, the present study is the first randomized placebo-controlled double-blind study investigating both short and long-term intradialytic CV effects of ARB in newly started HD patients. Due to equal BP-levels in the two treatment groups as intended, our study primarily provides results on BP-independent effects of RAAS-blockade. At equal BP-levels, there was no significant effect of ARB on intradialytic central hemodynamic parameters. IDH was not significantly different in the two groups over a one-year period. Moreover, our study yielded detailed one-year follow-up data on intradialytic central hemodynamics, which is novel.

Avoiding hemodynamic instability during HD is important because it hinders sufficient fluid removal, causes inadequate dialysis, and may add to CV risk [5, 36]. Thus, frequent episodes of IDH is suspected to cause myocardial [37, 38] and cerebral ischemia [39] and has been found to be related both to frontal lobe atrophy[40] and rise in cardiac biomarkers (troponin I and creatine kinase isoenzyme MB)[41]. The use of antihypertensive drugs might well influence the ability to adjust for changes in plasma volume, thereby contributing to IDH. Only few studies are available on the tolerability of antihypertensive drugs during HD [1, 2, 42–46], none of them being long-term, except for the study by Tisler et al.[2]. This ten-month observational cohort-study demonstrated that use of long-acting nitrates and absence of calcium channel blockers increased the risk of IDH, but also reported that patients with frequent IDH episodes were taking less antihypertensive medication compared to those with few IDH episodes. As noted by the authors, these findings probably reflect a consequence rather than a cause. Most likely due to a commonly held practice by physicians to withhold antihypertensive drugs in patients with frequent IDH episodes.

Other studies investigating intradialytic hemodynamic parameters have primarily focused on modifiable HD-related parameters such as dialysate calcium [47, 48] and sodium concentration [49], thermal effects [50], use of continuous blood volume monitoring [51, 52], and comparison of HD vs. hemodiafiltration [53, 54]. Several of these studies also used the saline dilution technique for assessment of CO [47, 50, 53, 54]. Our study showed that CO decreased during HD due to a reduction in SV, which was not fully compensated by an increase in HR. A similar intradialytic response was found in previous short-term studies [47, 50, 54]. The intradialytic response to HD was stable over a one-year period and intradialytic central hemodynamic parameters were overall unaffected by ARB treatment. CBV represents the relative blood volume responding to fluid removal by UF during HD [31, 53]. The small increase in CBV within both groups during the study period may reflect progressive volume overload as urine output decreased. Interestingly, this increase developed despite frequent clinical assessment of hydration status and despite reductions in BP.

On average, the ARB-treated group received two defined daily doses of antihypertensive medication (300 mg irbesartan) more than the placebo group, but both groups achieved a similar decrease in predialytic BP and the predefined mean BP-target of 140 mmHg was reached in both groups. Intradialytic MAP within the first and last 30 minutes of HD, as well as the intradialytic change in MAP were similar and overall constant in the two groups when all twelve months were taken into account. Beforehand, we expected a greater decrease in MAP and TPR during dialysis in ARB-treated patients due to the vasodilatory effect of irbesartan (blockade of the angiotensin type 1 (AT1) receptor). However, this response was not evident apart from ΔMAP at six and nine months and ΔTPR at six and twelve months, respectively. There is no obvious explanation for these findings except for the fact that BP was reduced equally in the placebo group. HD-time, UF volume, and use of additional antihypertensive medication were not significantly different. Moreover, CBV tended to be higher in the ARB group and predialytic weight tended to decrease in the placebo group and increase in the ARB group suggesting higher volume overload in the ARB group. Thus, we cannot exclude that subtle differences in volume control might have attenuated the additional BP-lowering effect of ARB. Whether more efficient volume control was achieved in the placebo group could potentially have been better clarified by use of more objective measures of volume status such as bioimpedance assessment [55, 56].

IDH was not significantly more frequent in ARB-treated patients in our study and overall there was no indication of significantly lower intradialytic BP in the ARB group. Our results corroborate findings by Davenport et al. who found no correlation between IDH and achievement of a predialytic BP-target of 140/90 mmHg, although achievement of a postdialytic BP-target of 130/80 was significantly correlated with symptomatic IDH episodes [1]. The same study also suggested that IDH was not made worse by the prescription of antihypertensive agents and that RAAS-blocking agents were not superior to other drug classes in terms of reaching the predialytic BP-target. Symptomatic IDH is reported to occur in 4–30% of HD treatments [1, 7, 9]. In our study, IDH episodes occurred infrequently, most likely due to the relatively low UF volumes prescribed. Moreover, we used a rather strict definition of IDH, which may have resulted in fewer events compared with previous studies. Our definition was based on recent recommendations in the European Best Practice Guideline, since IDH has not been standardized and differs between various studies [45]. Comorbid conditions known to affect regulatory CV mechanisms such as CV disease and diabetes were evenly distributed in the two groups. However, the relatively low number of patients in our study prevented adequately powered analysis regarding the effect of ARB in these subgroups.

Previous studies have shown that formation of an AV-fistula has a significant impact on CV parameters such as CO, TPR, BP, and SV [57, 58]. All intradialytic measurements in our study were performed in patients with an AV-fistula and our results cannot be extrapolated to HD patients with permanent venous catheters. CV instability during HD may be more prevalent among more morbid and fragile patients. These patients are unlikely participants in a one-year trial such as ours and absence of these patients will inevitably influence our results. Due to preserved urine output in the majority of our patients relatively small UF volumes were prescribed during HD compared to other studies [2, 5]. In HD patients with more pronounced CV disease or larger fluid fluctuations, the response to ARB treatment may differ. Thus, our results should be interpreted with some caution and our study cannot exclude the possibility that ARB treatment could be associated with adverse effects if given in doses reducing BP more than within the control group. Furthermore, it is possible that a higher-powered study could show significantly more IDH episodes. In this respect, our patients had remarkably few IDH episodes, which was excellent for the success of their treatment but less advantageous for the study power. Finally, our study was limited to a one-year follow-up period and a longer follow-up could perhaps reveal significant effects of ARB treatment.

In conclusion, the ARB irbesartan was well tolerated without significant side effects when given as an add-on to other antihypertensive therapy in HD patients with some residual renal function, a relatively short dialysis vintage and low ultrafiltration volumes. At equal BP-levels, central hemodynamics during dialysis was not significantly affected by ARB treatment. Low intradialytic BP was slightly more prevalent in ARB-treated patients, but this was not significant. Apart from an increase in CBV, there was no significant change in intradialytic CV parameters such as CO, SV, TPR, HR, and MAP over time. Whether anuric HD patients with longer HD-vintage and larger UF volumes behave similarly remains to be clarified.

## Supporting Information

S1 CONSORT Checklist (DOC)

S1 Dataset Zip-file archive containing: Dataset A (Demographics), Dataset B (Hemodynamics), and Dataset C (IDH logistic regression). (ZIP)

S1 Fig Intradialytic hemodynamics at start of dialysis. HD_START_: Intradialytic measurement within the first 30 minutes of dialysis; CO: Cardiac output; MAP: Mean arterial blood pressure; HR: Heart rate; TPR: Total peripheral resistance; SV: Stroke volume; CBV: Central blood volume (volume of blood in the heart, lungs, and the great vessels). Mean differences in CBV (baseline-12 months) were 0.1(0.0–0.2) L; *P* = 0.03 (Placebo) and 0.1(-0.1–0.2) L; *P* = 0.45 (ARB) at HD_START_. Comparison of the mean differences (baseline-12 months) at HD_START_ yielded a mean difference (Placebo vs. ARB) of 0.0(-0.2–0.13) L; *P* = 0.62. *) 0.05>*P*>0.01 vs. baseline within the placebo or ARB group
(TIFF)

S2 Fig Intradialytic hemodynamics at end of dialysis. HD_END_: Intradialytic measurement within the last 30 minutes of dialysis; CO: Cardiac output; MAP: Mean arterial blood pressure; HR: Heart rate; TPR: Total peripheral resistance; SV: Stroke volume; CBV: Central blood volume (volume of blood in the heart, lungs, and the great vessels). Mean differences in CBV (baseline-12 months) were 0.1(-0.0–0.3) L; *P* = 0.12 (Placebo) and 0.1(0.0–0.2) L; *P* = 0.03 (ARB) at HD_END_. Comparison of the mean differences (baseline-12 months) at HD_END_ yielded a mean difference (Placebo vs. ARB) of 0.0(-0.2–0.17) L; *P* = 0.85. *) 0.05>*P*>0.01 vs. baseline within the placebo or ARB group †) 0.05>*P*> 0.01 vs. placebo
(TIFF)

S1 Protocol Research protocol (Danish ver). (PDF)

S2 Protocol Appendix to research protocol (Danish ver). (PDF)

S3 Protocol Translation of the main points of the protocol. (DOC)

S1 Table Multivariate repeated measurements model (xtmixed) results. (DOCX)

S1 Text Secondary findings. (DOCX)

## References

[1] Davenport A, Cox C, Thuraisingham R. Achieving blood pressure targets during dialysis improves control but increases intradialytic hypotension. Kidney Int. 2008;73(6):759–64. 10.1038/sj.ki.5002745 PubMed . 18160959

[2] Tislér A, Akócsi K, Hárshegyi I, Varga G, Ferenczi S, Grosz M, et al Comparison of dialysis and clinical characteristics of patients with frequent and occasional hemodialysis-associated hypotension. Kidney Blood Press Res. 2002;25(2):97–102. 63515. PubMed . 12077491 10.1159/000063515

[3] Civati G, Guastoni C, Teatini U, Perego A, Perrino ML, Benazzi E, et al High-flux acetate haemodialysis: a single-centre experience. Nephrol Dial Transplant. 1991;6 Suppl 2:75–81. PubMed . 1866074

[4] al-Muhanna FA, Saeed I, al-Muelo S, Larbi E, Rubaish A. Disease profile, complications and outcome in patients on maintenance haemodialysis at King Faisal University Hospital, Saudi Arabia. East Afr Med J. 1999;76(12):664–7. PubMed . 10734534

[5] Shoji T, Tsubakihara Y, Fujii M, Imai E. Hemodialysis-associated hypotension as an independent risk factor for two-year mortality in hemodialysis patients. Kidney Int. 2004;66(3):1212–20. 10.1111/j.1523-1755.2004.00812.x PubMed . 15327420

[6] Sands JJ, Usvyat LA, Sullivan T, Segal JH, Zabetakis P, Kotanko P, et al Intradialytic hypotension: frequency, sources of variation and correlation with clinical outcome. Hemodial Int. 2014;18(2):415–22. 10.1111/hdi.12138 PubMed . 24467830

[7] Daugirdas JT. Pathophysiology of dialysis hypotension: an update. Am J Kidney Dis. 2001;38(4 Suppl 4):S11–7. PubMed . 11602456 10.1053/ajkd.2001.28090

[8] Daugirdas JT. Dialysis hypotension: a hemodynamic analysis. Kidney Int. 1991;39(2):233–46. PubMed . 2002637 10.1038/ki.1991.28

[9] Santoro A. Cardiovascular dialysis instability and convective therapies. Hemodial Int. 2006;10 Suppl 1:S51–5. 10.1111/j.1542-4758.2006.01192.x PubMed . 16441871

[10] van Der Sande FM, Kooman JP, Leunissen KM. Strategies for improving hemodynamic stability in cardiac-compromised dialysis patients. Am J Kidney Dis. 2000;35(5):E19 PubMed . 10793048 10.1016/s0272-6386(00)70284-8

[11] Barakat MM, Nawab ZM, Yu AW, Lau AH, Ing TS, Daugirdas JT. Hemodynamic effects of intradialytic food ingestion and the effects of caffeine. J Am Soc Nephrol. 1993;3(11):1813–8. PubMed . 8329676 10.1681/ASN.V3111813

[12] Brewster UC, Perazella MA. The renin-angiotensin-aldosterone system and the kidney: effects on kidney disease. Am J Med. 2004;116(4):263–72. 10.1016/j.amjmed.2003.09.034 PubMed . 14969655

[13] Timmermans PB, Wong PC, Chiu AT, Herblin WF, Benfield P, Carini DJ, et al Angiotensin II receptors and angiotensin II receptor antagonists. Pharmacol Rev. 1993;45(2):205–51. PubMed . 8372104

[14] Dahlof B, Devereux RB, Kjeldsen SE, Julius S, Beevers G, de FU, et al Cardiovascular morbidity and mortality in the Losartan Intervention For Endpoint reduction in hypertension study (LIFE): a randomised trial against atenolol. Lancet. 2002;359(9311):995–1003. 11937178 10.1016/S0140-6736(02)08089-3

[15] Pfeffer MA, McMurray JJ, Velazquez EJ, Rouleau JL, Køber L, Maggioni AP, et al Valsartan, captopril, or both in myocardial infarction complicated by heart failure, left ventricular dysfunction, or both. N Engl J Med. 2003;349(20):1893–906. 10.1056/NEJMoa032292 PubMed . 14610160

[16] Takahashi A, Takase H, Toriyama T, Sugiura T, Kurita Y, Ueda R, et al Candesartan, an angiotensin II type-1 receptor blocker, reduces cardiovascular events in patients on chronic haemodialysis—a randomized study. NephrolDialTransplant. 2006;21(9):2507–12. http://ndt.oxfordjournals.org/cgi/content/full/22/1/281. 16766543 10.1093/ndt/gfl293

[17] Suzuki H, Kanno Y, Sugahara S, Ikeda N, Shoda J, Takenaka T, et al Effect of angiotensin receptor blockers on cardiovascular events in patients undergoing hemodialysis: an open-label randomized controlled trial. AmJKidney Dis. 2008;52(3):501–6. 10.1053/j.ajkd.2008.04.031 18653268

[18] Cice G, Di Benedetto A, D'Isa S, D'Andrea A, Marcelli D, Gatti E, et al Effects of Telmisartan Added to Angiotensin-Converting Enzyme Inhibitors on Mortality and Morbidity in Hemodialysis Patients With Chronic Heart Failure A Double-Blind, Placebo-Controlled Trial. Journal of the American College of Cardiology. 2010;56(21):1701–8. 10.1016/j.jacc.2010.03.105 PubMed . 21070920

[19] Zannad F, Kessler M, Lehert P, Grünfeld JP, Thuilliez C, Leizorovicz A, et al Prevention of cardiovascular events in end-stage renal disease: results of a randomized trial of fosinopril and implications for future studies. Kidney Int. 2006;70(7):1318–24. 10.1038/sj.ki.5001657 PubMed . 16871247

[20] Iseki K, Arima H, Kohagura K, Komiya I, Ueda S, Tokuyama K, et al. Effects of angiotensin receptor blockade (ARB) on mortality and cardiovascular outcomes in patients with long-term haemodialysis: a randomized controlled trial. Nephrol Dial Transplant. 2013. 10.1093/ndt/gfs590. PubMed . 23355629

[21] Tai D, Lim T, James M, Manns B, Tonelli M, Hemmelgarn B, et al Cardiovascular effects of angiotensin converting enzyme inhibition or angiotensin receptor blockade in hemodialysis: a meta-analysis. Clin J Am Soc Nephrol. 2010;5(4):623–30. CJN.07831109 [pii] 10.2215/CJN.07831109 PubMed 20133488 PMC2849693

[22] Yang LY, Ge X, Wang YL, Ma KL, Liu H, Zhang XL, et al Angiotensin receptor blockers reduce left ventricular hypertrophy in dialysis patients: a meta-analysis. Am J Med Sci. 2013;345(1):1–9. 10.1097/MAJ.0b013e318249d387 PubMed . 23018492

[23] Knoll GA, Sahgal A, Nair RC, Graham J, van Walraven C, Burns KD. Renin-angiotensin system blockade and the risk of hyperkalemia in chronic hemodialysis patients. Am J Med. 2002;112(2):110–4. PubMed . 11835948 10.1016/s0002-9343(01)01068-3

[24] Agarwal R, Sinha AD, Pappas MK, Abraham TN, Tegegne GG. Hypertension in hemodialysis patients treated with atenolol or lisinopril: a randomized controlled trial. Nephrol Dial Transplant. 2014;29(3):672–81. 10.1093/ndt/gft515 PubMed 24398888 PMC3938300

[25] Peters CD, Kjærgaard KD, Jespersen B, Christensen KL, Jensen JD. Renal and cardiovascular effects of irbesartan in dialysis patients—a randomized controlled trial protocol (SAFIR study). Dan Med J. 2013;60(4):A4602 PubMed . 23651713

[26] Peters CD, Kjaergaard KD, Jensen JD, Christensen KL, Strandhave C, Tietze IN, et al No significant effect of angiotensin II receptor blockade on intermediate cardiovascular end points in hemodialysis patients. Kidney Int. 2014;86(3):625–37. 10.1038/ki.2014.69 PubMed . 24670413

[27] Kjaergaard KD, Peters CD, Jespersen B, Tietze IN, Madsen JK, Pedersen BB, et al. Angiotensin Blockade and Progressive Loss of Kidney Function in Hemodialysis Patients: A Randomized Controlled Trial. Am J Kidney Dis. 2014;[published online ahead of print July 8, 2014]. 10.1053/j.ajkd.2014.05.011 PubMed . 25011693

[28] Sica DA, Marino MR, Hammett JL, Ferreira I, Gehr TW, Ford NF. The pharmacokinetics of irbesartan in renal failure and maintenance hemodialysis. ClinPharmacolTher. 1997;62(6):610–8. 9433389 10.1016/S0009-9236(97)90080-1

[29] Marino MR, Langenbacher K, Ford NF, Uderman HD. Pharmacokinetics and pharmacodynamics of irbesartan in healthy subjects. J Clin Pharmacol. 1998;38(3):246–55. PubMed . 9549663 10.1002/j.1552-4604.1998.tb04422.x

[30] Krivitski NM. Novel method to measure access flow during hemodialysis by ultrasound velocity dilution technique. ASAIO J. 1995;41(3):M741–M5. 8573905 10.1097/00002480-199507000-00111

[31] Krivitski NM, Depner TA. Cardiac output and central blood volume during hemodialysis: methodology. AdvRen ReplaceTher. 1999;6(3):225–32. http://www.journals.elsevierhealth.com/periodicals/yjarr/article/S1073-4449(99)70018-X/abstract. 10452705 10.1016/s1073-4449(99)70018-x

[32] Kisloukhine VV, Dean DA. Validation of a novel ultrasound dilution method to measure cardiac output during hemodialysis. ASAIO J. 1996;42(5):M906–M7. 8945018 10.1097/00002480-199609000-00125

[33] Nikiforov YV, Kisluchine VV, Chaus NI. Validation of a new method to measure cardiac output during extracorporeal detoxification. Asaio Journal. 1996;42(5):M903–M5. PubMed . 8945017 10.1097/00002480-199609000-00124

[34] Tsutsui M, Matsuoka N, Ikeda T, Sanjo Y, Kazama T. Comparison of a New Cardiac Output Ultrasound Dilution Method With Thermodilution Technique in Adult Patients Under General Anesthesia. Journal of Cardiothoracic and Vascular Anesthesia. 2009;23(6):835–40. 10.1053/j.jvca.2009.03.007 PubMed PMID: WOS:000276965800013 19464193

[35] Moser M, Kenner T. Blood flow and blood volume determinations in aorta and in coronary circulation by density dilution. Basic Research in Cardiology. 1988;83(6):577–89. 10.1007/bf01906951 PubMed . 3223874

[36] Henderson LW. Symptomatic intradialytic hypotension and mortality: an opinionated review. Semin Dial. 2012;25(3):320–5. 10.1111/j.1525-139X.2012.01068.x PubMed . 22452289

[37] McIntyre CW, Burton JO, Selby NM, Leccisotti L, Korsheed S, Baker CS, et al Hemodialysis-induced cardiac dysfunction is associated with an acute reduction in global and segmental myocardial blood flow. ClinJAmSocNephrol. 2008;3(1):19–26. 10.2215/CJN.03170707 PMC2390980 18003765

[38] Dasselaar JJ, Slart RH, Knip M, Pruim J, Tio RA, McIntyre CW, et al Haemodialysis is associated with a pronounced fall in myocardial perfusion. NephrolDialTransplant. 2009;24(2):604–10. 10.1093/ndt/gfn501 18775808

[39] Toyoda K, Fujii K, Fujimi S, Kumai Y, Tsuchimochi H, Ibayashi S, et al Stroke in patients on maintenance hemodialysis: a 22-year single-center study. Am J Kidney Dis. 2005;45(6):1058–66. PubMed . 15957135 10.1053/j.ajkd.2005.02.028

[40] Mizumasa T, Hirakata H, Yoshimitsu T, Hirakata E, Kubo M, Kashiwagi M, et al Dialysis-related hypotension as a cause of progressive frontal lobe atrophy in chronic hemodialysis patients: a 3-year prospective study. Nephron ClinPract. 2004;97(1):c23–c30. 15153764 10.1159/000077592

[41] Hung SY, Hung YM, Fang HC, Yeh JH, Hung GC, Wu CJ, et al Cardiac troponin I and creatine kinase isoenzyme MB in patients with intradialytic hypotension. Blood Purif. 2004;22(4):338–43. 10.1159/000079188 PubMed . 15218282

[42] Sherman RA, Casale P, Cody R, Horton MW. Effect of predialysis verapamil on intradialytic blood pressure in chronic hemodialysis patients. ASAIO Trans. 1990;36(2):67–9. PubMed . 2340210 10.1097/00002480-199004000-00005

[43] Davenport A, Cox C, Thuraisingham R. Blood pressure control and symptomatic intradialytic hypotension in diabetic haemodialysis patients: a cross-sectional survey. Nephron Clin Pract. 2008;109(2):c65–71. 10.1159/000139991 PubMed . 18560240

[44] Man in 't Veld AJ, Schicht IM, Derkx FH, de Bruyn JH, Schalekamp MA. Effects of an angiotensin-converting enzyme inhibitor (captopril) on blood pressure in anephric subjects. Br Med J. 1980;280(6210):288–90. PubMed 6986949 10.1136/bmj.280.6210.288 PMC1600124

[45] Kooman J, Basci A, Pizzarelli F, Canaud B, Haage P, Fouque D, et al EBPG guideline on haemodynamic instability. Nephrol Dial Transplant. 2007;22 Suppl 2:ii22–44. 10.1093/ndt/gfm019 PubMed . 17507425

[46] Leidig M, Bambauer R, Kirchertz EJ, Szaba T, Handrock R, Leinung D, et al Efficacy, safety and tolerability of valsartan 80 mg compared to irbesartan 150 mg in hypertensive patients on long-term hemodialysis (VALID study). ClinNephrol. 2008;69(6):425–32. 18538118 10.5414/cnp69425

[47] Karamperis N, Sloth E, Jensen JD. The hemodynamic effect of calcium ion concentration in the infusate during predilution hemofiltration in chronic renal failure. Am J Kidney Dis. 2005;46(3):470–80. 10.1053/j.ajkd.2005.05.022 PubMed . 16129209

[48] van der Sande FM, Cheriex EC, van Kuijk WH, Leunissen KM. Effect of dialysate calcium concentrations on intradialytic blood pressure course in cardiac-compromised patients. Am J Kidney Dis. 1998;32(1):125–31. PubMed . 9669433 10.1053/ajkd.1998.v32.pm9669433

[49] van Kuijk WH, Wirtz JJ, Grave W, de Heer F, Menheere PP, van Hooff JP, et al Vascular reactivity during combined ultrafiltration-haemodialysis: influence of dialysate sodium. Nephrol Dial Transplant. 1996;11(2):323–8. PubMed . 8671787 10.1093/oxfordjournals.ndt.a027261

[50] Karamperis N, Jensen D, Sloth E, Jensen JD. Comparison of predilution hemodiafiltration and low-flux hemodialysis at temperature-controlled conditions using high calcium-ion concentration in the replacement and dialysis fluid. ClinNephrol. 2007;67(4):230–9. 17474559 10.5414/cnp67230

[51] Gabrielli D, Krystal B, Katzarski K, Youssef M, Hachache T, Lopot F, et al Improved intradialytic stability during haemodialysis with blood volume-controlled ultrafiltration. J Nephrol. 2009;22(2):232–40. PubMed . 19384841

[52] Barth C, Boer W, Garzoni D, Kuenzi T, Ries W, Schaefer R, et al Characteristics of hypotension-prone haemodialysis patients: is there a critical relative blood volume? Nephrol Dial Transplant. 2003;18(7):1353–60. PubMed . 12808173 10.1093/ndt/gfg171

[53] Beerenhout C, Dejagere T, van der Sande FM, Bekers O, Leunissen KM, Kooman JP. Haemodynamics and electrolyte balance: a comparison between on-line pre-dilution haemofiltration and haemodialysis. Nephrol Dial Transplant. 2004;19(9):2354–9. 10.1093/ndt/gfh315 PubMed . 15266029

[54] Karamperis N, Sloth E, Jensen JD. Predilution hemodiafiltration displays no hemodynamic advantage over low-flux hemodialysis under matched conditions. Kidney Int. 2005;67(4):1601–8. 15780117 10.1111/j.1523-1755.2005.00242.x

[55] Hur E, Usta M, Toz H, Asci G, Wabel P, Kahvecioglu S, et al Effect of Fluid Management Guided by Bioimpedance Spectroscopy on Cardiovascular Parameters in Hemodialysis Patients: A Randomized Controlled Trial. Am J Kidney Dis. 2013;61(6):957–65. 10.1053/j.ajkd.2012.12.017 PubMed . 23415416

[56] Daugirdas JT. Bioimpedance technology and optimal fluid management. Am J Kidney Dis. 2013;61(6):861–4. 10.1053/j.ajkd.2013.03.004 PubMed . 23684491

[57] Korsheed S, Eldehni MT, John SG, Fluck RJ, McIntyre CW. Effects of arteriovenous fistula formation on arterial stiffness and cardiovascular performance and function. Nephrol Dial Transplant. 2011. gfq851 [pii] 10.1093/ndt/gfq851 PubMed . 21317408

[58] Basile C, Lomonte C, Vernaglione L, Casucci F, Antonelli M, Losurdo N. The relationship between the flow of arteriovenous fistula and cardiac output in haemodialysis patients. NephrolDialTransplant. 2008;23(1):282–7. 17942475 10.1093/ndt/gfm549

