# Supplementary figures and images for: Short and Long-Term Effects of the Angiotensin II Receptor Blocker Irbesartan on Intradialytic Central Hemodynamics: A Randomized Double-Blind Placebo-Controlled One-Year Intervention Trial (the SAFIR Study)
Source: PLoS One. 2015 Jun 1;10(6):e0126882. doi: 10.1371/journal.pone.0126882 (PMC4452642; doi:10.1371/journal.pone.0126882)

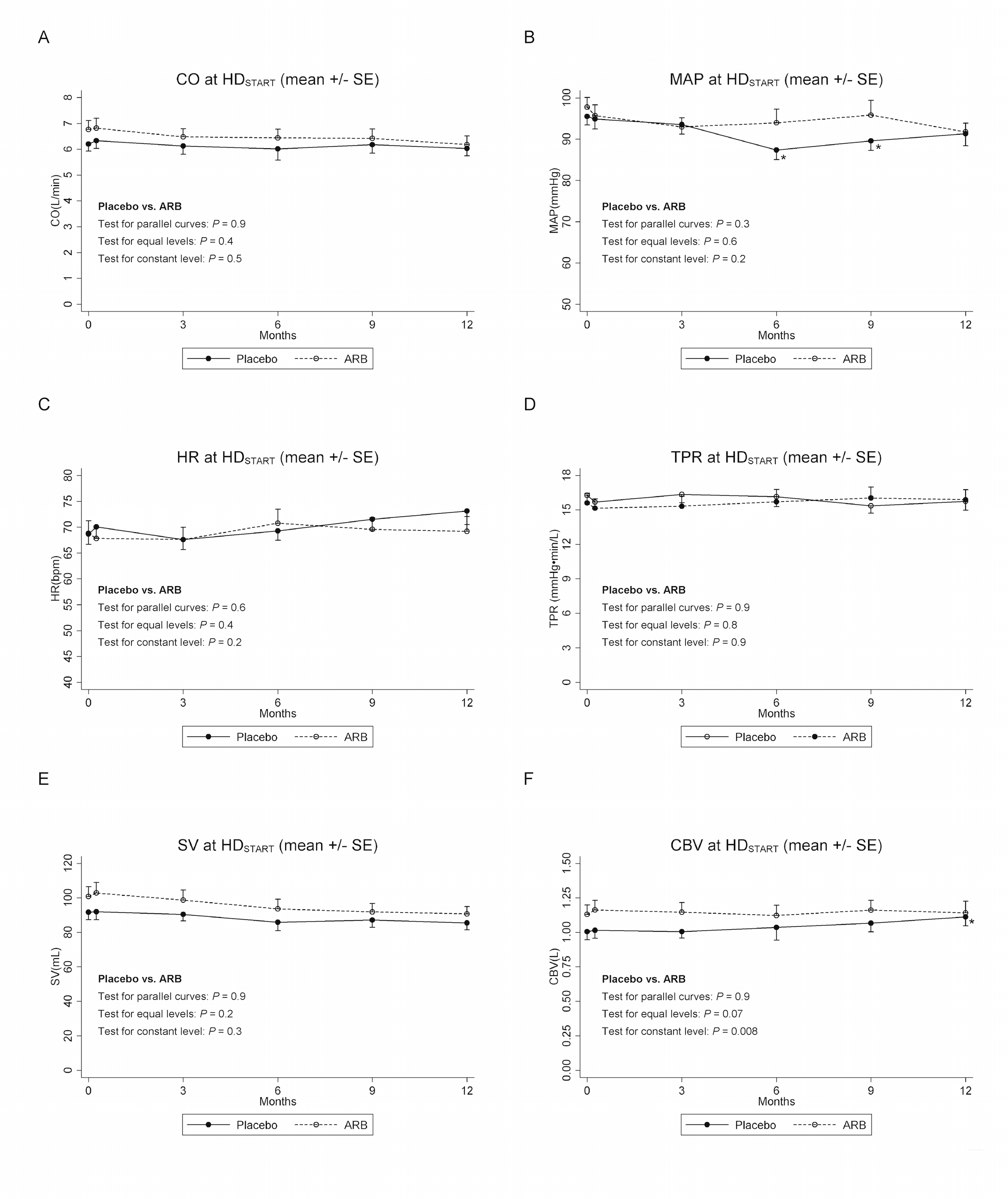

Supplement: S1 Fig — HDSTART: Intradialytic measurement within the first 30 minutes of dialysis; CO: Cardiac output; MAP: Mean arterial blood pressure; HR: Heart rate; TPR: Total peripheral resistance; SV: Stroke volume; CBV: Central blood volume (volume of blood in the heart, lungs, and the great vessels). Mean differences in CBV (baseline-12 months) were 0.1(0.0–0.2) L; P = 0.03 (Placebo) and 0.1(-0.1–0.2) L; P = 0.45 (ARB) at HDSTART. Comparison of the mean differences (baseline-12 months) at HDSTART yielded a mean difference (Placebo vs. ARB) of 0.0(-0.2–0.13) L; P = 0.62. *) 0.05>P>0.01 vs. baseline within the placebo or ARB group (TIFF) [file pone.0126882.s003.tiff]

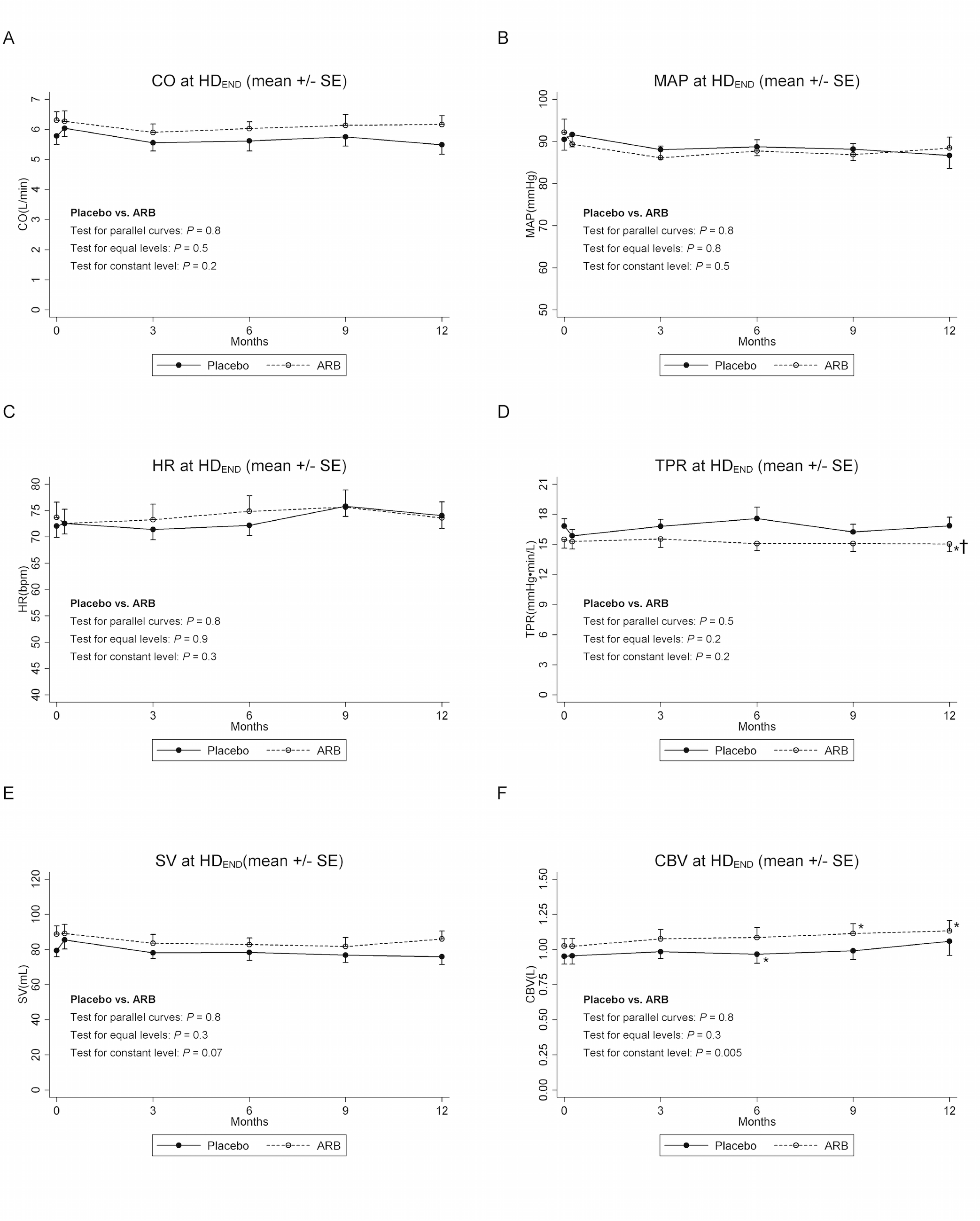

Supplement: S2 Fig — HDEND: Intradialytic measurement within the last 30 minutes of dialysis; CO: Cardiac output; MAP: Mean arterial blood pressure; HR: Heart rate; TPR: Total peripheral resistance; SV: Stroke volume; CBV: Central blood volume (volume of blood in the heart, lungs, and the great vessels). Mean differences in CBV (baseline-12 months) were 0.1(-0.0–0.3) L; P = 0.12 (Placebo) and 0.1(0.0–0.2) L; P = 0.03 (ARB) at HDEND. Comparison of the mean differences (baseline-12 months) at HDEND yielded a mean difference (Placebo vs. ARB) of 0.0(-0.2–0.17) L; P = 0.85. *) 0.05>P>0.01 vs. baseline within the placebo or ARB group †) 0.05>P> 0.01 vs. placebo (TIFF) [file pone.0126882.s004.tiff]
