# Supplementary material for: Short and Long-Term Effects of the Angiotensin II Receptor Blocker Irbesartan on Intradialytic Central Hemodynamics: A Randomized Double-Blind Placebo-Controlled One-Year Intervention Trial (the SAFIR Study)
Source: PLoS One. 2015 Jun 1;10(6):e0126882. doi: 10.1371/journal.pone.0126882 (PMC4452642; doi:10.1371/journal.pone.0126882)
Supplement: S1 Protocol — (PDF) [file pone.0126882.s005.pdf]

Bevarelse af restnyrefunktion hos hæmodialysepatienter ved behandling med en angiotensin II antagonist – en dobbeltblind randomiseret undersøgelse

PROJEKTGRUPPE

Krista Dybtved Kjærgaard[1,2], cand.med.

Christian Daugaard Peters[1], cand.med.

Jens Kristian Dam Jensen[1], lektor, ph.d., overlæge (sponsor-investigator)

Charlotte Strandhave[3], cand.med., klinisk assistent

Ida Tietze[4], ph.d., overlæge

Marija Kristina Novosel[5], cand.med.

Inge Eidemak[7], ph.d., overlæge

Erik Sloth[6], dr.med., overlæge

Bente Jespersen[1,2], professor, dr.med., overlæge

Fra

[1] Nyremedicinsk afdeling C, Århus Universitetshospital, Skejby

[2] Klinisk Institut, Århus Universitet

[3] Nyremedicinsk Afdeling, Medicinerhuset, Aalborg Sygehus Syd

[4] Medicinsk afdeling M, Regionshospitalet Viborg

[5] Medicinsk afdeling, Fredericia Sygehus

[6] Anæstesiologisk og Intensiv afdeling I, Århus Universitetshospital, Skejby

7 Nefrologisk klinik, Afsnit 2132, Rigshospitalet

Forsøgsansvarlig samt autoriseret til at udføre opgaver for sponsor jvf. fuldmagt til dette, se appendiks:

Krista Dybtved Kjærgaard, tlf. 8949 5706 / 2232 4321

RESUMÉ

Baggrund: Praksis er tilbageholdenhed med brug af ACE-hæmmer/angiotensin II receptor blokker (ARB), når patienter når dialysestadiet, idet stofferne akut kan nedsætte glomerulær filtrationshastighed (GFR). 2 studier blandt asiatiske P-dialysepatienter har vist, at en ACE-hæmmer og en ARB har signifikant effekt på bevarelse af restnyrefunktionen. Samtidig har studier vist, at bevarelse af restnyrefunktion blandt hæmodialysepatienter har stor betydning for livskvalitet, sygelighed og dødelighed.

Formål: Vi ønsker primært at undersøge, om irbesartan (en ARB) kan mindske tabet af restnyrefunktion blandt danske hæmodialysepatienter. Sekundært undersøger vi irbesartans effekter på hjerte og kar, tiden til udvikling af komplet anuri og udvikling i selvrapporteret livskvalitet.

Design: Dobbeltblindet, placebo-kontrolleret randomiseret multicenterforsøg med deltagelse af dialysecentre i Aalborg, Fredericia, Viborg, Skejby, Randers og Horsens samt på Rigshospitalet og Frederiksberg Hospital. 80 patienter skal påbegynde projektmedicin mhp. forventet gennemførelse for 60 patienter. Hver patient følges i et år.

Undersøgelser: Oversigt over undersøgelserne findes i appendiks. Primær effektparameter er tab af restnyrefunktion. Desuden undersøges hjerte og kar vha. ekkokardiografi, applanationstonometri og Transonic-måling af cardiac output. Livskvaliteten undersøges med dialysepatient-specifikt spørgeskema: KDQOL-SF.

Medicin: Patienterne randomiseres til placebo eller irbesartan. Projektmedicinen optitreres til 300 mg * 1 dagligt under hensyntagen til evt. bivirkninger. Alle involverede parter er blindet ift. hvilke patienter, der modtager hvilken medicin. Koden brydes, når undersøgelsen er afsluttet.

Økonomi: Undersøgelsen er forsker-initieret og finansieres ved hjælp af fondsmidler. Projektmedicinen leveres af medicinalproducenten Sanofi-Aventis.

INDHOLDSFORTEGNELSE

Formål 4
Baggrund 4
Hypoteser 6
Design 6
  Forsøgspersoner 7
  Inklusionskriterier 7
  Eksklusionskriterier 7
  Kriterier for udtrædelse af forsøget 8
  Opfølgning ved drop out eller eksklusion 8
  Kriterier for at stoppe forsøget 8
Undersøgelser 9
  Undersøgelser ved inklusion 9
  Baseline værdier 9
  Opfølgning undervejs i projektet 10
  Undersøgelser, månedskontrol 11
  Undersøgelser, kvartalskontrol 12
  Nyrefunktionsbestemmelse 13
  Blodtryk 14
  Kalium 14
  Ekkokardiografi 14
  Applanationstonometri 15
  Hjerte-frekvens-variabilitet (HRV) 15
  Transonic-bestemmelse af cardiac output 16
  KDQOL-SF 16
Biobank 16
Randomiseringsprocedure 17
Projektmedicin 17
  Irbesartan 18
  Placebo 18
  Dosering af projektmedicin 18
  Bivirkninger og sikkerhed 19
Undersøgelsens varighed 22
Statistik 22
Praktiske muligheder for at gennemføre projektet 23
Etik 24
Honorering for deltagelse 26
Dataregistrering 26
Forsikring 27
Økonomi 28
Publikation 28
Referenceliste 29

Appendiks:
Skema over undersøgelser, livskvalitets-spørgeskema (KDQOL-SF), lægmandsresumé, rekrutteringsbrev, deltagerinformation, samtykkeerklæring, fuldmagtserklæring, deltagerkort, autorisation fra sponsor til koordinerende investigator, produktresumé for irbesartan, information om placebo (IMPD), kontrakt mellem sponsor og Sanofi-Aventis, tillæg til kontrakt.

FORMÅL

Vi ønsker at undersøge om behandling med en angiotensin II antagonist kan føre til bevarelse af restnyrefunktionen hos hæmodialysepatienter. Desuden vil vi vurdere, om der er andre sidegevinster ved behandlingen i form af bedre hjerte-karfunktion i forhold til, hvad der udvikles under et års dialyse i en tilsvarende gruppe patienter behandlet med placebo.

BAGGRUND

Når patienter påbegynder dialyse, har de ofte en restnyrefunktion svarende til 10-15 % af det normale. Man har tidligere ikke fokuseret så meget på bevarelse af denne funktion, men snarere på at optimere dialysen, som giver en rensning svarende til ca. 10 % af normal nyrefunktion. Det har nu imidlertid vist sig, at restnyrefunktionen betyder mere end dialysen for patientens sygelighed og dødelighed[1,2]. Desuden har bevarede diureser stor betydning for livskvaliteten, da væskerestriktion er et hovedproblem for de fleste dialysepatienter.

Nyrefunktion kan hos såvel raske som syge kvantiteres ved glomerulær filtrations rate (GFR), som hos raske typisk er 80-120 ml/min. Når GFR < 20 ml/min, er patienten i prædialyse-stadiet, hvor der kommer mange komplikationer til den svigtende nyrefunktion (nedsat vandudskillelse, ophobning af affaldsstoffer, forskydning af salte i blodet, forstyrret knoglemetabolisme, nedsat produktion af erytropoietin, evt. tab af protein via urinen). De fleste har behov for dialyse ved GFR < 10 ml/min. De fleste patienter har blodtryksforhøjelse allerede før dette stadie.

Restnyrefunktionen aftager oftest hurtigt i månederne efter indledning af dialyse. Diureser kan dog presses lidt op ved at holde patienten i en konstant overhydreret tilstand, som på længere sigt belaster hjertet.

Der foreligger kun ganske sparsom forskning vedrørende hæmodialysepatienters bevarelse af restnyrefunktion, hvorfor området er dårligt belyst og med modstridende data. Mange faktorer menes involveret i tabet af restnyrefunktion: demografiske faktorer (inkluderende alder, køn, race), tilgrundliggende nyresygdom, blodtrykskontrol, proteinuri, nefrotoksiske medikamina, blodtrykssænkende medicin (angiotensin converting enzyme inhibitor (ACEI)/angiotensin II receptor blokker (ARB)), komorbiditet, akut sygdom og kronisk inflammation.[1] Blodtryksfald

under dialysen fører formentlig til hurtigere tab af restnyrefunktion, og det vides ikke, om disse episoder forværres under behandling med ACEI/ARB, hvilket vil kunne modvirke den nyrebeskyttende effekt, som kendes før dialysestadiet. Intradialytiske hypotensive hændelser kan reduceres ved anvendelse af "Blood Volume Monitor" (BVM), som registrerer blodvolumen kontinuerligt under dialyse og kan sættes til at alarmere, hvis en grænseværdi nås eller eventuelt direkte styre ultrafiltrationen. På den måde kan væske-elimination under dialysen tilpasses patientens evne til at mobilisere sit væskeoverskud til blodbanen, og derved kan for hurtigt væsketræk med faldende blodtryk til dels undgås.[3]

I P-dialysepopulationen er der påvist en række potentielle fordele ved bevaret restnyrefunktion i form af bedre clearance af toksiner med mellemstore og store molekylvægte, bedre volumen- og blodtrykskontrol, mindre inflammation, øget appetit og ernæringsstatus, relativ bevarelse af nyrernes endokrine funktioner, øget fosfatkontrol samt øget livskvalitet.[1]

De samme faktorer må antages til en vis grad gøre sig gældende blandt hæmodialysepatienter.

Dialysepatienter har en kardiovaskulær dødelighed, der i de yngre aldersklasser er mere end 100 gange større end hos jævnaldrende raske[4], og ved ekkokardiografi findes oftest hjertehypertrofi, mens karrene findes stive førende til hurtig pulsbølgehastighed og ændret pulskvalitet, som kan registreres ved en enkel og non-invasiv metode kaldet applanationstonometri, fx SphygmoCor®, der netop er blevet valideret hos patienter med uræmi[5].
Hjerte-frekvens-variabilitet (HRV) er nedsat hos mange dialysepatienter, hvilket er vist at være en markør for øget mortalitet.[6] HRV kan undersøges vha. Holter-monitorering, men kan også estimeres vha. SphygmoCor® apparaturet nævnt ovenfor. Nogle dialysepatienter bliver nyretransplanterede, men også denne gruppe patienter har en væsentlig overdødelighed af hjerte-kar-sygdom, som formentlig kan nedbringes ved bevarelse af restnyrefunktion i nødvendige dialyseperioder, da nyresvigt via til dels ukendte mekanismer forårsager accelereret udvikling af kardiovaskulær sygdom.

Diabetikere har øget forekomst af såvel mikro- som makrovaskulære forandringer med øget sygelighed og dødelighed til følge, og op mod 20-30 % af incidente dialysepatienter i Danmark har diabetes.[7]

Der foreligger to studier af P-dialysepatienters restnyrefunktion under behandling med hhv. ACEI i 12 måneder[8] og ARB i 1 år og 9 måneder[9], mens præparaternes effekt på hæmodialysepatienters restnyrefunktion ikke er belyst. Studierne af P-dialysepatienter har vist en gavnlig effekt på bevarelse af restnyrefunktionen sammenlignet med placebo. I begge studier er restnyrefunktionen kvantiteret ved hjælp af døgnurinopsamling og efterfølgende bestemmelse af kreatinin- og/eller carbamidudskillelsen. Denne undersøgelse anvendes i Danmark rutinemæssigt til vurdering af restnyrefunktion, og metoden er derfor velkendt for dialysepatienterne.

HYPOTESER

1) Behandling med ARB kan bevare restnyrefunktion hos hæmodialysepatienter (primær effektparameter)
2) Behandlingen med ARB kan bevare eller forbedre hjerte-kar-funktionen bedømt ved ekkokardiografi og applanationstonometri. ARB giver forskel i antallet af alvorlige kardiovaskulære hændelser og død (sammensat endepunkt) samt forlænger tiden til udvikling af komplet anuri (døgnurinvolumen < 100 ml). ARB giver mindre forværring i livskvalitet bedømt ved KDQOL-SF (sekundære effektparametre).

DESIGN

Patienter, som påbegynder eller har påbegyndt hæmodialysebehandling indenfor det seneste år, og som opfylder nedenstående inklusionskriterier, anmodes om at indgå i projektet. Der inkluderes patienter, indtil 80 patienter er påbegyndt behandling med projektmedicin.
Ved inklusion randomiseres forsøgspersonerne til enten aktiv behandling i form af irbesartan eller placebo. Patienterne randomiseres i to linjer afhængigt af diabetes-status, og der blokrandomiseres inden for hvert center. Randomiseringen skal være dobbelt-blindet under hele studiet.
Patienterne kontrolleres herefter hver anden uge i to måneder og derpå månedligt på lokalt dialysecenter til sikring af samme blodtryksniveau og undgåelse af hyperkaliæmi. Hver 3. måned undersøges nyrefunktionen samt en række andre parametre – se skema i appendiks. Hver patient følges i et år.

Forsøgspersoner

For at nå op på 80 patienter inden for en inklusionsperiode på godt 2 år, vil vi rekruttere patienter fra sygehusene i Ålborg, Fredericia og Viborg, Randers og Horsens samt Rigshospitalet og Frederiksberg Hospital. Det anslås, at 60 patienter fuldfører projektet.

Inklusionskriterier

- Hæmodialysepatient
- Startet hæmodialyse inden for de seneste 12 mdr
- Bevaret restnyrefunktion med døgnurinvolumen > 300 ml
- Informeret samtykke
- Alder > 18 år
- Antikonception i form af p-piller/spiral/plaster/ring/implantat/gestagen-injektion, hvis patienten er en fertil kvinde

Eksklusionskriterier

- Systolisk blodtryk < 110 mm Hg
- Dårlig almentilstand efter skøn
- Patienter, som ikke vil kunne forstå formålet
- Kendt allergi over for ACE-hæmmer/ARB
- Akut myokardieinfarkt eller ustabil angina pectoris inden for de seneste 3 mdr
- Tidligere myokardieinfarkt med nedsat venstre ventrikelfunktion (ejection fraction = EF < 30 %) med eller uden symptomer på hjerteinsufficiens
- Hjerteinsufficiens på non-iskæmisk basis med nedsat venstre ventrikelfunktion (EF < 30 %)
- Positiv graviditetstest (testen udføres, såfremt patienten er menstruerende)

Selv om patienten undervejs i projektet udvikler restnyrefunktionstab, således at døgnurinvolumen falder til < 300 ml, så fortsætter behandlingen og undersøgelsesprogrammet uden ændringer.

Kriterier for udtrædelse af forsøget

- Vedvarende hyperkaliæmi > 6 mmol/l trods diæt, Resonium og dosisreduktion af projektmedicin
- AMI (STEMI/nonSTEMI) diagnosticeret på klassisk vis ved 2 ud af 3 af følgende: brystsmerter/dyspnø, klassiske EKG-forandringer, forhøjelse af biokemiske markører
- Hjertesvigt diagnosticeret i anden sammenhæng med EF < 30 %
- Angioødem med formodet relation til projektmedicinen
- Nyretransplantation
- Tilbagetrækning af samtykke til deltagelse i projektet
- Patienten overholder ikke aftaler og/eller kan ikke samarbejde omkring undersøgelser

Patienterne ophører med projektmedicinen i forbindelse med eksklusion.

Opfølgning ved drop out eller eksklusion

Ved eksklusion eller drop out efter mere end 3 måneders deltagelse i projektet foretages om muligt applanationstonometri (evt. inkl. HRV), døgnurinopsamling, EKG, Transonic og ekkokardiografi, og spørgeskema vedr. livskvalitet udfyldes. Hvis patienten udgår af studiet pga. bivirkninger, skal patienten følges for at sikre, at bivirkningerne igen forsvinder. Hvis generne består i mere end 1 måned efter ophør med projektmedicinen, kan årsags-virknings-sammenhængen mellem projektmedicin og bivirkninger betvivles, og patienten overgår til kontrol i vanligt hæmodialyseafsnit.
Data for den enkelte patient vil indgå i databehandling og analyse, såfremt patienten har gennemført inklusionsbesøg og 3 måneders besøg.
Alle eksklusioner vil blive dokumenteret i patientens case report forms (CRF) og medicinske journal med information vedrørende eventuelle komplikationer, bivirkninger eller andre årsager til udtrædelse.

Kriterier for at stoppe forsøget

Forsøget stoppes i sin helhed før tid, hvis der fremkommer ny viden, som tyder på, at det er uforsvarligt at anvende irbesartan til hæmodialysepatienter.

UNDERSØGELSER

En oversigt over alle undersøgelser er vedlagt i appendiks.

Undersøgelser ved inklusion

Hvis patienten udtrykker velvilje ift. deltagelse i projektet, skal vedkommende have foretaget ekkokardiografi samt døgnurinopsamling for at sikre, at døgnurinvolumen > 300 ml.
Hvis EF < 30 %, da henvises patienten til kardiologisk vurdering og kan ikke indgå i projektet qua eksklusionskriterierne. Hvis EF < 30 % , og patienten er over- eller dehydreret, kan optimering af hydreringsgrad forsøges før fornyet ekkokardiografi. Hvis systolisk blodtryk < 110 mm Hg, og patienten får antihypertensiv medicin uden kardiologisk indikation, da nedtrappes/seponeres medicinen mhp. at bringe systolisk blodtryk > 110 mm Hg.
Såfremt patienten på inklusionstidspunktet modtager ACE-hæmmer eller ARB på ikke-kardiologisk indikation, stopper vedkommende med behandlingen, og efter wash out periode på 1 uge (T½ = 11-15 timer)[10] foretages nyrefunktionsundersøgelse 1. gang. Herefter kan behandling med projektmedicin indledes, og alle patienter får gentaget nyrefunktionsundersøgelsen efter ca. en uges behandling. Både ved baseline og efter 1 uge undersøges patienterne med undersøgelsespakken hørende til 3 måneders kontroller – se nedenfor.

Baseline værdier

Baggrundsoplysninger: Fødselsdato, køn, højde, rygerstatus, race, primær nyresygdom, komorbiditet, nyretransplantation med angivelse af årstal, evt. fjernede native/transplanterede nyrer samt dato for første dialyse.
Målinger: Vægt, blodtryk ved dialysestart (manuelt samt gennemsnit fra den sidste uges dialyser), puls, blodprøver (kalium, natrium, kreatinin, carbamid, kuldioxid/standard-bikarbonat, albumin, fosfat, calcium, hæmoglobin, lipider (total-kolesterol, LDL, HDL, triglycerider), ALAT/ASAT, basisk fosfatase, bilirubin, PTH, CRP, hgbA1c, p-glukose, cystatin C, NT-proBNP, angiotensin II, aldosteron, renin, adrenalin, noradrenalin, inflammationsmarkører), nyrefunktionsbestemmelse, applanationstonometri, Transonic-bestemmelse af cardiac output, U-albumin, U-kreatinin, U-carbamid, U-natrium, U-kalium, U-steroidmetabolitter, døgnurinvolumen, Kt/V (værdi for dialyse-intensitet), EKG, hjerte-frekvens-variabilitet (HRV) i en subpopulation, og diabetikere skal

medbringe blodsukkerprofil fra 3 døgn med blodsukker fastende, før-middag, før-aftensmad og kl. 22. Livskvaliteten bedømmes kvantitativt vha. spørgeskemaet KDQOL-SF.
Der optages grundig anamnese, især mhp. vanlige gener, fx svimmelhed, hovedpine, muskelspændinger for at kunne differentiere mellem habituel tilstand og evt. medicin-bivirkninger, ligesom objektiv undersøgelse gennemføres (se nedenfor).
Desuden skal der foretages ekkokardiografi.

Opfølgning undervejs i projektet

Patienterne kontrolleres initialt hver 2. uge, især med henblik på justering af blodtryk og kontrol af plasma kalium. Efter 2 måneder overgår patienten til kontrol efter behov, men mindst én gang månedlig +/- 1 uge. Disse konsultationer foregår på lokalt hæmodialyseafsnit.
På hvert dialysecenter vil der være en ansvarlig læge samt en tilknyttet projektsygeplejerske til koordinering af undersøgelser, blodprøvebestilling samt medicinrekvirering.
Nyrefunktionsbestemmelse, applanationstonometri (evt. inkl. HRV), EKG og Transonic-bestemmelse af cardiac output udføres ved 3, 6, 9 og 12 måneder +/- 2 uger.
Spørgeskemaet angående livskvalitet besvares efter 6 og 12 måneder +/- 2 uger, og ekkokardiografi samt applanationstonometri dagen efter en dialyse udføres efter 11,5 - 12 måneder.
Der udføres afsluttende besøg efter, at projektmedicin er seponeret.

Objektiv undersøgelse, udføres i forbindelse med første besøgsdag

- Øjne
- Mund
- Hjerte og lunger
- Abdomen
- Lymfeknudestatus
- Hud

Undersøgelser, månedskontrol

I CRF noteres følgende ved hver projekt-relateret kontakt mellem patient og læge:

- Vægt
- Blodtryk ved dialysestart – gennemsnit fra den sidste uges dialyser
- Puls
- Hydreringsstatus ud fra klinisk vurdering
- Medicinliste
- Dialyse:
  - Ønskevægt
  - Væsketræk – gennemsnit fra den sidste uges dialyser
  - Dialyseform
  - Dialysemængde
  - Filtertype og størrelse
  - Flow – gennemsnit fra den sidste uges dialyser
  - Buffer Ca^{++}
  - Anvendelse af ultrarent vand
  - Antal registrerede blodtryksfald siden sidst
    - Laveste blodtryk
    - Behandling af blodtryksfaldet
    - Konsekvenser for dialyseordinationer
- Indlæggelse
  - Afdeling
  - Tidsrum
  - Årsag
  - Projektmedicin taget som foreskrevet under indlæggelsen
- I interviewform udspørges til:
  - Åndenød
  - Brystsmerter/hjertebanken
  - Compliance mht. medicin generelt og projektmedicin i særdeleshed
  - Mistanke om bivirkninger
  - Andre subjektive forandringer siden sidste kontakt
  - Større undersøgelser siden sidst, især med brug af røntgenkontrast-midler

Laboratorieværdier, taget ved dialysestart, vedlægges CRF i udskrift:

- o Kalium
- o Natrium
- o Kreatinin
- o Carbamid
- o Kuldioxid/standard-bikarbonat
- o Albumin
- o Fosfat
- o Calcium

Undersøgelser, kvartalskontrol

I CRF noteres:

- Blodtryk – måling med godkendt apparat
- Døgnurinvolumen

CRF vedlægges data om nedenstående i udskrift eller certificeret kopi:

- Diabetikere medbringer blodsukkerprofil fra 3 døgn med blodsukker fastende, før-middag, før-aftensmad og kl. 22.
- Nyrefunktionsbestemmelse
- Applanationstonometri
- HRV – hvis muligt
- Transonic-bestemmelse af cardiac output
- EKG
- KDQOL-SF ved baseline samt efter 6 og 12 måneder
- Blodprøver:
    - o Hæmoglobin
    - o HgbA1c
    - o Glukose
    - o ALAT/ASAT, basisk fosfatase, bilirubin
    - o Lipider (total-cholesterol, LDL, HDL, triglycerider)
    - o PTH
    - o Cystatin C

  - CRP
  - NT-proBNP
  - Aldosteron
  - Renin
  - Angiotensin II (EVF-standardiseret)
  - Noradrenalin
  - Adrenalin
  - Inflammationsmarkører
  - Prøver tages fra til senere forskningsbrug
- Urinprøver
  - Albumin
  - Kreatinin
  - Carbamid
  - Natrium
  - Kalium
  - Steroidmetabolitter
  - Prøver tages fra til senere forskningsbrug
- Kt/V (værdi for dialyse-intensitet)

Undersøgelsespakken hver 3. måned varetages af investigator i samarbejde med projektsygeplejerske, ligesom månedskontroller kan udføres delvist af projektsygeplejerske.
Hvis en patient viser sig at være fuldstændig stabil mht blodtryk, vægt og hydrering, kan investigator ved kontrolbesøg overveje at erstatte den kliniske vurdering af hydreringsgrad med telefonkonsultation eller overlade hydreringsvurderingen til trænet sygeplejerske. Hydrering skal altid vurderes af læge ifm kvartalsbesøg.
Investigator behøver ikke nødvendigvis at udføre den lægelige del af kontrol og besøg på den samme dag som den ikke-lægelige del.

Nyrefunktionsbestemmelse

Nyrefunktionen kan vurderes ud fra estimat af GFR. I klinisk dagligdag anvendes kreatinin-clearance baseret på døgnurinopsamling. I dette studie anvendes gennemsnittet af kreatinin-clearance og carbamid-clearance, da præliminære data fra et studie ledet af Krista Dybtved

Kjærgaard indikerer, at kreatinin-carbamid-clearance udviser større reproducerbarhed end kreatinin-clearance. Urinen skal af dialysepatienten opsamles minutiøst gennem 24 timer op til dialysestart.[11] NSAID skal undgås den sidste uge op til nyrefunktionsundersøgelse pga. risiko for forbigående reduktion i GFR.[12]

Blodtryk

Da blodtryksniveauet må forventes at have en selvstændig indvirkning på de målte effektparametre, søges dette holdt på et bestemt niveau hos patienterne, der indgår i undersøgelsen, idet der tilstræbes systolisk blodtryk[13] 135-140 mm Hg ved dialysestart ved hjælp af projektmedicin og sædvanlige antihypertensive midler andre end ACEI, ARB og renin-hæmmer, samt ved væskeelimination efter sædvanlige principper. Hos de få patienter, der har blodtryk lavere end dette niveau, og som er uden symptomer, accepteres det lave blodtryk.
Mindst en gang månedligt foretages ”diagnostisk blodtryksmåling” med godkendt apparat/dialyseapparat (hvis disse kalibreres), hvilket er beskrevet i Dansk Hypertensionsselskabs guideline.[14]

Kalium

Pga. den begrænsede nyrefunktion og pågående dialysebehandling forventes ikke ud fra kliniske erfaringer eller tidligere studie[15] større problemer med plasma kalium, men hvis værdierne ved dialysestart er $\geq$ 6,0 mmol/l, indledes diætetiske forholdsregler, ordination af Resonium overvejes, og dosis af ARB/placebo kan reduceres.
Hvis plasma kalium vedvarende er $\geq$ 6,0 mmol/l, udgår patienten af forsøget.

Ekkokardiografi

Ved ekkokardiografien vurderes hjertets dimensioner i form hulrums-mål (atrium/ventrikel-dilatation) og hjertemusklens vægtykkelse (ventrikelhypertrofi) samt hjertet pumpefunktion i form EF, som hos raske > 60 %. Desuden kan klapproblemer, fx i form af forsnævringer og forkalkninger, og anatomiske abnormiteter visualiseres, hvilket svarer til en basal ekkokardiografi. Alle ekkokardiografier optages således, at det senere vil være muligt at lave mere avanceret analyse af data.

Der foretages ekkokardiografi ved inklusion og ved udgang af studiet – så vidt muligt inden seponering af projektmedicin. Begge gange skal undersøgelsen laves dagen efter en dialyse for at sikre mest mulig sammenlignelighed mellem patienterne.

Applanationstonometri

Applanationstonometri består af to dele: pulsbølgeanalyse og pulsbølgehastighedsmåling.
Applanationstonometrien foretages med SphygmoCor® (AtCor Medical), som også er anvendt i andre studier og er valideret blandt uræmi-patienter.[5]
Undersøgelsen foretages på perifere arterier (a. carotis, a. radialis, a. femoralis) med måling af tryk overført fra arteriens væg til transducer. Undersøgelsen er non-invasiv og uden gener for patienten.
Pulsbølgeanalyse:
For hvert hjerteslag dannes en trykbølge, som forplanter sig i hele arterietræet. Når trykbølgen møder en forandring i modstand, vil der ske en refleksion af bølgen. Disse bølger danner tilsammen en karakteristisk bølgeform, som transmitteres til software i SphygmoCor. Bølgeformen ændres med alder, vasodilatation, vasokonstriktion og tiltagende karstivhed.
Pulsbølgehastighed:
Aortas pulsbølgehastighed er et direkte mål for aortas stivhed, som er en stærk uafhængig prædiktor for kardiovaskulær død og død i det hele taget blandt patienter med kronisk nyresvigt.[16]
Undersøgelsen er koblet til EKG og måler derfor tiden mellem afsendelse af trykbølge fra hjertet (EKG'ets R-tak) til registrering af trykbølge ved transducer. Afstanden måles manuelt, hvorefter hastigheden kan beregnes.
Undersøgelsen varer i alt ca. ½ time og udføres umiddelbart før dialyse ved inklusion og derefter hver 3. måned. Der laves desuden applanationstonometri dagen efter en dialyse ved udgang af studiet.

Hjerte-frekvens-variabilitet (HRV)

Ved hjælp af special-software i SphygmoCor apparatet og de tilhørende EKG-elektroder kan pulsen registreres over et tidsrum, oftest ca. 5 minutter. Software beregner relevante variable vedr. variation i frekvens sv.t. Holter-monitorering. Undersøgelsen udføres på patienter i Skejby, Randers, Horsens, Aalborg og Viborg og om muligt også på de øvrige centre.

Transonic-bestemmelse af cardiac output

Med Transonic Systems Inc.'s HD02/03 kan cardiac output og centralt blodvolumen estimeres ved en fortyndingsteknik vha. specielle sensorer, som kobles til dialyseslangerne.
Cardiac output måles inden for den første halve time efter dialysestart samt ved dialyseslut ved inklusion og ved 3, 6, 9 og 12 måneder. Undersøgelsen gennemføres på patienter, som dialyseres i Skejby, Aalborg, Fredericia, Viborg, Randers og Horsens og om muligt også på Rigshospitalet og Frederiksberg Hospital.

KDQOL-SF

Der findes talrige spørgeskemaer, som kan anvendes til at bedømme helbreds-relateret livskvalitet. I KDQOL-SF er der taget hensyn til de specifikke problemer, som dialysepatienter kan have i form af afhængighed af sygehusvæsenet, væskerestriktion, diæt med mere. Spørgeskemaet er valideret.[17,18]
Spørgeskemaet udfyldes af patienten ved inklusion samt ved 6 og 12 måneder. Patientens svar omsættes til kvantitative værdier for livskvalitet, hvorved livskvaliteten kan sammenlignes til forskellige tidspunkter for den samme patient og sammenlignes i de to behandlingsgrupper.

BIOBANK

I forbindelse med projektet undersøges blod og urin, som opbevares i en forskningsbiobank fra udtagelse fra patienten frem til undersøgelsestidspunktet. Der vil ved hver kvartals-kontrol blive udtaget ca. 50 ml blod til senere analyse for Cystatin C, NT-proBNP, renin, angiotensin II, aldosteron, adrenalin og noradrenalin. Der er ingen risiko forbundet med indsamlingen af biologisk materiale, da blodprøverne tages ifm. rutineprøver. Alle analyser forventes at kunne udføres i Danmark, men nogle af analyserne er relativt kostbare og kan med økonomisk fordel analyseres samlet efter projektets afslutning, hvorfor det biologiske materiale gemmes indtil analyse eller op til 2 år efter forsøget afslutning, hvorefter materialet anonymiseres. Fra den opsamlede døgnurin gemmes 50 ml til analyse for steroidmetabolitter.
Oprettelsen af denne forskningsbiobank anmeldes til Videnskabsetisk Komité samt Datatilsynet.
Der vil ligeledes blive gemt blod og urin, opbevaret i personhenførbar form mhp. senere forskningsprojekter, såfremt patienten tillader dette. Dette anmeldes alene til Datatilsynet.

Randomiseringsprocedure

Medicinen randomiseres til henholdsvis Skejby inkl. Randers og Horsens, Aalborg, Fredericia, Rigshospitalet, Frederiksberg Hospital og Viborg formentlig i blokke samt i serier til diabetikere og til ikke-diabetikere. Hospitalsapoteket Aarhus vil stå for randomiseringen, som foretages med "the method of randomly permuted blocks", opbevaring af kodelister samt pakning og etikettering af medicin, hvilket vil blive gjort i overensstemmelse med gældende retningslinjer for "Good Manufacturing Practice"[19]

Patienterne inkluderes fortløbende og får udleveret medicin i henhold til fortløbende numre.

Når den sidste patient har været til afsluttende kontrol, brydes randomiseringen. Indtil da er randomiseringen dobbelt-blindet, således at hverken patient eller involveret sundhedsfagligt personale ved, om patienten har fået irbesartan eller placebo.

Hospitalsapoteket leverer en kuvert pr. forsøgsnummer indeholdende information vedrørende projektmedicinen. Kuverterne opbevares i aflåst rum på de involverede dialysecentre, og kuverterne er sikret mod utidig indsigt i randomisering.

Randomiseringskoden kan brydes i tilfælde af eksklusion af en deltager, hvor information vedrørende forsøgsmedicinen er nødvendig for optimal behandling af patienten. Hospitalsapoteket informeres om bruddet, og der indsamles nødvendig information vedrørende den administrerede forsøgsmedicin. Alle relevante hændelser og information vedrørende kodebrud vil blive registreret.

Projektmedicin

I klinisk praksis har næsten alle prædialysepatienter forhøjet blodtryk, og hos mange bliver blodtrykket sværere og sværere at regulere, efterhånden som nyrefunktionen aftager. En del prædialysepatienter får ACEI/ARB dels pga. disse præparaters kendte renoprotektive effekter og dels som antihypertensiva. Efterhånden som dialysegrænsen nærmer sig, får mange patienter problemer med hyperkaliæmi, hvorfor ACEI/ARB seponeres. Desuden er man tilbageholdende med antihypertensiv medicin efter dialysestart, idet blodtryksfald under dialysen typisk forværres af medikamenterne. Senere genoptages antihypertensiv behandling efter behov. Der er ikke tradition for at bruge ACEI eller ARB som 1.valgs præparat til hæmodialysepatienter, og der er således ikke tale om, at patienten "snydes" for gængs behandling.

Hvis patienten ved inklusionen får ACEI eller ARB, skal patienten stoppe med dette, og efter en wash out periode på en uge bestemmes restnyrefunktionen. Patientens eventuelle behov for at opstarte ny medicin eller få øget dosis af vanlige antihypertensivae beror på et individuelt klinisk

skøn, hvor der tilstræbes systolisk blodtryk ved dialysestart på 135 - 140 mm Hg. Et højere blodtryk kan accepteres efter individuelt lægefagligt skøn.

Irbesartan

Medikamentet er beskrevet i produktresuméet, se appendiks.[20]

Placebo

Placebotabletterne ligner irbesartan 150 mg fuldstændigt, men indeholder selvfølgelig ingen aktive substanser. En nærmere beskrivelse af tabletterne (IMPD) kan ses i appendiks.

Dosering af projektmedicin

Projektmedicinen gives efter følgende retningslinjer[20]:
Initialt: 150 mg * 1 dagligt
Optitrering: Efter 2 uger øges dosis til 300 mg * 1 dagligt
Vedligeholdelsesdosis: 300 mg * 1 dagligt, hvilket er anbefalet max dosis.

Patienterne skal optitreres til maksimal anbefalet dosis uanset blodtryksniveauet. Det vil sige, at hvis patienten har lavt blodtryk og er uden symptomer på dette, fastholdes behandlingen. Andre antihypertensiva reduceres eller seponeres som anført nedenfor. Hvis der efter gentagne forsøg på optitrering opstår symptomatisk hypotension, da accepteres sub-maksimal dosis, og hvis symptomgivende hypotension da fortsat er et problem, udgår patienten af forsøget.
Hver dosisændring anføres på særskilt skema.
Ved hypertension skal patientens hydreringsgrad vurderes, og ved overhydrering skal ønskevægten reduceres efter sædvanlige principper, hvorefter patientens antihypertensive behandling kan øges. Alle præparater på nær renin-hæmmere, ACEI og ARB kan anvendes.
NSAID-behandling skal undgås i ugen op til nyrefunktionsbestemmelsen (kvartals-kontrol).
God compliance tilstræbes ved at spørge til tablet-indtag i forbindelse med kvartalskontrol. Dette sammenholdes med antallet af udleverede/tilbageleverede tabletter. Projektsygeplejersker vil ligeledes opmuntre patienterne og give gode råd til at huske medicinen, f.eks. i form af ophældning i dosisæsker sammen med øvrig medicin.

Projektet er planlagt som placebo-kontrolleret, randomiseret og dobbelt-blindet studie, da dette anses for at være det stærkeste design, når der skal afprøves lægemidler.[21]
Irbesartan- og placebotabletter skal være af ens størrelse, farve, lugt og smag for at hindre skævt frafald f.eks. på grund af grimtsmagende tabletter. Sundhedspersonalet må desuden ikke på baggrund af tabletternes fysiske egenskaber eller indpakning kunne gennemskue, om patienten får irbesartan eller placebo.
Hospitalsapoteket Aarhus vil stå for pakning af projektmedicinen, som leveres af medicinalproducenten Sanofi-Aventis. Medicinen leveres løbende til de enkelte centre.
Forsøgsmedicinen skal opbevares i aflåst rum, og der vil blive holdt regnskab med antallet af udleverede og returnerede tabletter i henhold til procedurer for ”good clinical practice”.

Bivirkninger og sikkerhed

Bivirkning	Forslag til handling
Symptomatisk hypotension	• Undgå hypovolæmi<br>• Samtidig behandling med diuretika og/eller vasodilatatorer reduceres om muligt<br>• Evt. forsøge at fordele døgndosis i morgen- og aftendosis (150 mg * 2)<br>• Hvis ovenstående ikke er muligt, skal dosis reduceres
Hyperkaliæmi (kalium > 6,0 mmol/L)	• Kontrol af kalium inden for 1 uge<br>• Reducér/seponér kaliumtilskud og kaliumbesparende diuretika<br>• Diætetisk vejledning<br>• Overveje ordination af Resonium<br>• Ved kalium > 6,0 trods ovenstående tiltag, da forsøges dosisreduktion til 150 mg dagligt/seponering af projektmedicin<br>• Når kalium ≤ 5,5 mmol/L, kan dosisøgning forsøges<br>• Ved vedvarende kalium > 6,0 mmol/L før dialyse, skal patienten udgå
Hovedpine, svimmelhed	• Sandsynligvis et resultat af blodtryksreduktion, derfor se venligst under ”hypotension” for behandlingsvejledning. Væskeindtagelsen kan reduceres mhp. at nedbringe væsketræk under dialyse

Bivirkningsfrekvensen er ikke undersøgt specifikt for hæmodialysepatienter.
I de undersøgte befolkningsgrupper, herunder diabetikere og hypertonikere er de hyppigste bivirkninger:
> 10 %: hyperkaliæmi
1-10 %: træthed, kvalme, opkastning, myalgi og svimmelhed.
Hvis patienten er i behandling med lithium, skal serum-lithiumniveauet kontrolleres, da irbesartan kan øge serum-lithiumkoncentrationen.[20]

Monitorering af hændelser

Alle uønskede hændelser håndteres og rapporteres i henhold til alle relevante regler og medtages i den endelige, kliniske forsøgsrapport. Hændelser, der opstår i præ- og postbehandlingsperioder, skal også indsamles. Rapportering og indsamling af uønskede hændelser skal derfor starte fra det tidspunkt, hvor en patient er indgået i forsøget (fra informeret samtykke er underskrevet), og indtil sidste besøg er overstået.

Definition af en uønsket hændelse (AE) og en alvorlig uønsket hændelse (SAE)

En hændelse er enhver uønsket klinisk tilstand, der forekommer hos en patient eller en forsøgsperson, som har modtaget et lægemiddel, og som ikke nødvendigvis behøver at have en årsagssammenhæng med denne behandling.
En alvorlig hændelse er enhver uønsket klinisk tilstand, der uanset dosis:

- Medfører døden eller
- Er livstruende*
- Kræver indlæggelse eller forlængelse af en igangværende indlæggelse eller
- Medfører vedvarende eller betydelig invaliditet/uarbejdsdygtighed eller
- Er en medfødt anomali/misdannelse.
- Er en klinisk betydende hændelse#:

*Betegnelsen "livstruende" i definitionen af "alvorlig" henfører til en hændelse, hvor patienten var i umiddelbar livsfare, da hændelsen forekom.
Eksempler på sådanne hændelser er intensiv behandling på en skadestue eller i hjemmet af allergisk bronkospasme, bloddyskrasier, kramper eller asymptomatisk forhøjelse af ALT til ≥10 ULN, der ikke medfører indlæggelse, eller udvikling af stofafhængighed eller stofmisbrug.

#Der skal foretages en lægelig og videnskabelig vurdering for at afgøre, om fremskyndet rapportering er påkrævet i andre situationer, som f.eks. betydende kliniske hændelser der ikke nødvendigvis er umiddelbart livstruende eller medfører døden eller indlæggelse, men som kan bringe patienten i fare eller kræve indgreb for at forebygge et af de andre udfald nævnt i ovenstående definition.

Alvorlige hændelser, der ikke skal håndteres som en SAE

- Planlagte hospitalsindlæggelser for behandling af eksisterende sygdom, som ikke er signifikant forværret.
- Hospitalsindlæggelse på grund af diagnostiske procedurer.
- Indlæggelser som alene vedrører dialyse-adgangsveje

Registrering af uønskede hændelser

Ved hver kontakt med patienten skal der aktivt spørges ind til forekomst af uønskede hændelser siden sidste besøg. Alle informationer om uønskede hændelser skal registreres i Case report formen. Alvorlige uønskede hændelser skal ligeledes registreres på SAE skemaet.
Alle uønskede hændelser skal følges indtil ophør af hændelse, stabilisering eller indtil det er afgjort, at forsøgslægemidlet eller deltagelse i forsøget ikke har været årsag til hændelsen.

Rapportering af alvorlige uønskede hændelser

Information om alle alvorlige uønskede hændelser skal indsamles og registreres på SAE skemaet. Alle SAE'er skal rapporteres til koordinerende investigator (Krista Dybtved Kjærgaard, 22 32 43 21) eller sponsor-investigator (Jens Dam Jensen, 89 49 57 77) senest førstkommende arbejdsdag efter, at investigator har fået kendskab til SAE'en. Oplysninger kan faxes på fax: 89 49 60 03.

Bivirkninger

En bivirkning er enhver skadelig og uønsket reaktion på et forsøgslægemiddel uanset dosis.

Rapportering af alvorlige uventede bivirkninger (SUSARs)

SAE'er, som vurderes til at have kausal sammenhæng med forsøgslægemidlerne, er alvorlige bivirkninger. De alvorlige bivirkninger, som er uventede (dvs. ikke er beskrevet i det godkendte produktresume for forsøgslægemidlerne), kaldes alvorlige uventede bivirkninger (Suspected unexpected serious adverse reactions).

SUSARs skal rapporteres til Lægemiddelstyrelsen inden for følgende tidsfrister:
Dødsfald eller livstruende SUSARs inden for 7 dage
Øvrige SUSARs inden for 15 dage

Sponsor-investigator (koordinerende center) eller dennes stedfortræder skal sende en kopi af de SUSARs, som rapporteres til Lægemiddelstyrelsen, til de deltagende forsøgsafdelinger og til Sanofi-Aventis, bivirkningsafdelingen (fax: 45 16 70 30).

Årlig sikkerhedsopdatering

En gang om året indsendes en sikkerhedsopdatering (ASR) til Lægemiddelstyrelsen og den Videnskabsetiske komité med en samlet oversigt over alle alvorlige uønskede hændelser (SAE), alle alvorlige bivirkninger (SAR) og alle alvorlige uventede bivirkninger (SUSAR) samt en vurdering af forsøgspersonernes sikkerhed. Kopi af sikkerhedsopdateringen sendes til Sanofi-Aventis' bivirkningsafdeling (fax: 45 16 70 30).

UNDERSØGELSENS VARIGHED

1. patient blev inkluderet primo april 2009, og inklusion forventes afsluttet 31.06.2011 med forventet afslutning af forsøget i efteråret 2012.

STATISTIK

Det har i andre studier med patientantal, som det planlagte, været muligt at påvise forskelle på bevarelse af restnyrefunktion blandt P-dialysepatienter. Det har også i studier af tilsvarende størrelse været muligt at vise forskelle i patienternes hjertefunktion vurderet ved ekkokardiografi og karfunktion vha. pulsbølgekvalitet og hastighed.[22]

Hvis patienter udgår undervejs vil deres resultater blive analyseret, såfremt de har gennemgået undersøgelser ved start og efter 3 måneder. Det forventes, at indledning af ARB vil føre til en reversibel reduktion af GFR og urinvolumen, som fastlægges ved undersøgelse umiddelbart før og en uge efter påbegyndt behandling. Herefter vil faldhastigheden for GFR udtrykt som ml/min/måned og urinvolumen udtrykt som ml/døgn/måned blive fastlagt i de fire 3 måneders perioder og for hele perioden og sammenlignet mellem de to grupper ved hjælp af t-test eller non-parametrisk test. Der vil også blive anvendt mere avanceret statistik som regressionsanalyse og ANOVA med gentagne målinger. Måling og registrering af data vil ske løbende, mens endelig analyse af effektparametre først vil gennemføres efter sidste patients afsluttende besøg.

I et tidligere studie, hvor GFR estimeredes ved hjælp af døgnurinopsamling blandt P-dialysepatienter med GFR-range 2,00-7,87 ml/min/1,73 m^2, fandt man, at restnyrefunktionen i

kontrolgruppen faldt med 3 ml/min/1,73 m^2/år. Patienterne havde i gennemsnit været i dialyse 10 måneder ved inklusionen[8] Da faldhastigheden er størst lige efter dialysestart, regner jeg med et fald på 4 ml/min/1,73 m^2/år. Standarddeviation (SD) på faldhastigheden er projektgruppen bekendt ikke beskrevet, men foreløbig analyse af et reproducérbarhedsstudie, som ledes af Krista Dybtved Kjærgaard, giver formodning om, at SD maksimalt er 1,725.
Med type-1 fejls risiko på 5 % (risiko for fejlagtigt at forkaste H_0 = signifikansniveau), type-2 fejls risiko på 20 % (risiko for fejlagtigt at acceptere H_0 svarende til teststyrke på 80 %), SD som anført ovenfor 1,725 ml/min/1,73 m^2 og mindste relevante difference 1,4 ml/min/1,73 m2 (= miredif sv.t. 35 % reduktion af forventet tab af restnyrefunktion), skal der anvendes i alt 48 forsøgspersoner.

PRAKTISKE MULIGHEDER FOR AT GENNEMFØRE PROJEKTET

Krista Dybtved Kjærgaard har i kraft af sin ansættelse som klinisk assistent løn fra Århus Universitet og vil være koordinerende investigator og dermed stå for den praktiske kliniske gennemførelse af projektet bistået af ph.d. studerende Christian Daugaard Peters. Bevilling fra Det Frie Forskningsråd sikrer løn til Kjærgaard og Peters frem til sommeren 2012.
Den nødvendige ekspertise, fysiske rammer, applanationstonometer, laboratorieforhold og patientgrundlag er tilgængelig inden for Århus Universitetshospital, Skejby, hvor der også er truffet aftale om gennemførelse af ekkokardiografi. Mhp. overholdelse af inklusionsperiode på to år udvides projektet til en multicenterundersøgelse, hvor Århus Universitetshospital Aalborg Sygehus, Fredericia Sygehus, Rigshospitalet, Frederiksberg Hospital samt Regionshospitalet Viborg har accepteret at deltage.
Hovedvejleder for ph.d. studiet er professor Bente Jespersen, som har alsidig forskningserfaring. Sponsor Jens Dam Jensen er leder af dialyseafdelingen på Nyremedicinsk afdeling C, Århus Universitetshospital, Skejby og har opnået væsentlig erfaring inden for det aktuelle forskningsfelt, specielt dialysepatienter og hæmodynamik. Jens Dam Jensen og Bente Jespersen har tidligere sammen publiceret artikler, herunder resultater fra en interventionsundersøgelse blandt dialysepatienter. Erik Sloth og Jens Dam Jensen har sammen undersøgt forskellige hæmodialyse-modaliteters kardiovaskulære effekter.
Charlotte Strandhave har ansættelse som klinisk assistent ved Aalborg Sygehus pr. 1. september 2008 og har tidligere deltaget i forskningsrelateret samarbejde, bl.a. inklusion og undersøgelse af projektpatienter.

Ida Tietze har skrevet ph.d. inden for renal reservekapacitet blandt raske, hypertonikere samt nyresyge og arbejder nu som overlæge på Regionshospitalet Viborg.
Marija Kristina Novosel er ansat i introduktionsstilling og forventer at fortsætte i hoveduddannelsesstilling inden for nefrologi. Marija Kristina Novosel har publiceret 2 artikler.
Inge Eidemak er overlæge tilknyttet hæmodialyseafsnittet på Rigshospitalet samt Frederiksberg Hospital og har bred erfaring indenfor kliniske studier af hæmodialysepatienter.
Gruppen har erfaring med gennemførelse af undersøgelser, der følger GCP-principper.
Forsøget udføres i overensstemmelse med protokollen, og de gældende myndighedskrav samt lovgivning vil blive overholdt.

ETIK

Etiske problemstillinger: Gevinsten ved forsøget forventes at være påvisning af irbesartans gavnlige effekter i form af bevarelse af restnyrefunktionen. Gevinsten skal vurderes i forhold til bivirkninger og risici, som består i mulige medicinbivirkninger til irbesartan, ekstra blodprøver, tidsforbrug samt besvær med opsamling af døgnurin.
Bivirkninger til irbesartan (hyppigste bivirkninger er hyperkaliæmi, svimmelhed og træthed) er sjældent af alvorlig sværhedsgrad og svinder for dosisreduktion.
Blodprøver tages i forbindelse med dialysestart, så patienterne ikke skal stikkes ekstra.
Vanligvis får ikke-uræmiske patienter med EF < 40 % ordineret en række medikamina mod hjerteinsufficiens. Patogenesen ved hjertesygdom hos uræmikere er ikke velbeskrevet, og det vides ikke om den samme intervention vil være gavnlig eller måske forværre tilstanden. Da ekkokardiografi ikke foretages rutinemæssigt blandt incidente hæmodialysepatienter, har vi intet kendskab til f.eks. EF i denne population, men vi har en formodning om, at EF er nedsat hos en del af vore patienter. Vi ønsker at undersøge irbesartans effekter på så stor en andel af de incidente hæmodialysepatienter som muligt af hensyn til den eksterne validitet og finder det forsvarligt at inkludere patienter med EF ned til 30 %, da der ikke foreligger studier på området.
For den enkelte patient er fordelene en lidt tættere kontrol end vanligt samt chancen for at få et potentielt gavnligt medikament.
Halvdelen af patienterne vil få placebo-medicin. Der findes ikke medikamenter, som anvendes med renoprotektivt sigte blandt hæmodialysepatienter, og patienterne snydes derfor ikke for gængs behandling, da det ikke vides, om medicinen har gavnlige effekter blandt hæmodialysepatienter.

Patienter med forhøjet blodtryk vil fortsat tilbydes behandling, blot med andre antihypertensivae end ACE-hæmmer/ARB/renin-hæmmer, og patienterne løber således ingen blodtryksmæssig risiko ved at deltage i studiet.
Anvendelsen af placebo er nødvendig for at give studiet maksimal styrke, da observerede virkninger ellers ikke med sikkerhed kan tilskrives medicinen.
Samlet set vurderes projektet at være videnskabsetisk forsvarligt.
Anmeldelse og godkendelse: Undersøgelsen forudsætter godkendelse af den regionale videnskabsetiske komité, idet Region Midtjylland er anmeldelsessted.
Informeret samtykke: Deltagelse i undersøgelsen forudsætter informeret skriftligt samtykke efter både mundtlig og skriftlig information. Den mundtlige information gives primært af den undersøgelsesansvarlige eller investigator på lokalt hæmodialyseafsnit, men kan også gives af dialysesygeplejerske eller projektsygeplejerske, som på forhånd er grundigt indført i projektets mål og metoder.
Patienten får udleveret eller tilsendt rekrutteringsbrev med vedlagt deltagerinformation inkl. rettigheder som patient i medicinsk forsøg samt samtykke- og fuldmagtserklæring.
Hæmodialysepatienten kontaktes herefter personligt i forbindelse med dialyse. Hvis patienten er positivt indstillet, får han/hun den mundtlige information i tilknytning til dialysen. Der vil her være lejlighed til at stille spørgsmål. Patienten får betænkningstid og kontaktes efter aftale personligt eller pr. telefon med henblik på deltagelse i projektet, typisk inden for 1 uge. Ved behov aftales tid til fornyet samtale, evt. med bisidder, hvorefter informeret samtykke kan gives umiddelbart eller ved førstkommende dialyse.

Alle informationssamtaler vil som udgangspunkt foregå i samtalerum, hvor der er mulighed for at lukke døren. Hvis patienten foretrækker samtale under dialysen, vil dette blive imødekommet. Erfaring med arbejde med hæmodialysepatienter tilsiger, at patienterne ofte ønsker samtale under dialysen for at undgå at skulle bruge ekstra tid på sygehuset.
Hæmodialysepatienter er generelt præget af svær kronisk sygdom og har ofte brug for at få information gentaget flere gange, ligesom de sjældent har overskud til at tage initiativ til f.eks. at ringe tilbage til investigator angående deltagelse i forsøg. Ovenstående procedure er valgt på baggrund af dette.
Patienten vil blive informeret om projektets formål og metoder samt ulemper og risiko. Patienten gøres opmærksom på, at deltagelse er fuldstændig frivillig, at man kan blive informeret igen med

deltagelse af en bisidder efter eget valg, og at man når som helst kan afbryde sin deltagelse, uden at dette får konsekvenser for den videre behandling.
Patienten kan udover at spørge den forsøgsansvarlige få adgang til flere oplysninger om projektet hos kontaktperson professor Bente Jespersen, tlf.nr.: 89 49 57 04.

HONORERING FOR DELTAGELSE

Deltagerne vil ikke blive honoreret for deltagelse i forsøget.

Umiddelbar nytte af deltagelse

Patienten bliver fulgt nøje med en række undersøgelser, som også anvendes i daglig klinisk praksis (blodprøver, urinopsamling, vægt, blodtryk), men i denne undersøgelse under meget kontrollerede former, hvilket kan betyde hurtigere og mere effektiv justering af "skæve" laboratorieværdier, overhydrering og hypertension.
Patienten får chance for at modtage en mulig potent behandling (irbesartan).
Hjertet bliver undersøgt med ekkokardiografi, og ved svært hjertesvigt (som ellers ikke nødvendigvis vil være opdaget) henvises patienten til kardiologisk vurdering.

Ubehag og risici

Forsøgspersonens gener i forbindelse med forsøget er tidsforbrug og besvær med opsamling af døgnurin, hvilket dog alligevel skal gøres jævnligt som grundlag for tilrettelæggelse af dialysebehandlingen.
Ved applanationstonometri skal patienten tilbringe en ½ time mere på sygehuset, ligesom patienten skal møde på sygehuset 2 gange på ikke-dialyse-dage mhp. ekkokardiografi.
Patienten skal modtage ekstra medicin i form af projektmedicinen. Der er sjældent væsentlige bivirkninger til irbesartan, og disse, som måtte opstå, svinder for dosisreduktion eller evt. seponering.[20]

DATAREGISTRERING

Den forsøgsansvarlige skal føre og gemme en liste indeholdende forsøgsnumre, fulde navne, adresser og telefonnumre over alle inkluderede forsøgspersoner. Data indsamles af investigatorer og projektsygeplejersker ved hjælp af patientjournaler (tidligere sygdomme og medicinfortegnelser i

det omfang, patienten ikke selv kan redegøre for dette), interview, kliniske og biokemiske undersøgelser og gemmes i CRF som enten håndskrevne data eller vedlagte udskrifter/certificerede kopier. Data vil blive overført til en forskningsdatabase til videre analyse og dokumentation af resultater. Dataoverførsel og computeranalyse vil håndteres strengt fortroligt. Forsøgspersonernes identitet vil forblive anonym, og der vil ikke være adgang til information for nogen tredje part, bortset fra den lovgivende myndighed, Lægemiddelstyrelsen og GCP-enhederne.
Projektets gennemførelse, datadokumentation og rapportering vil blive foretaget i overensstemmelse med protokollen, ICH GCP guidelines og gældende lovgivning. GCP-enhederne og Lægemiddelstyrelsen har autoritet til at monitorere, foretage audits og inspektion af projektet.
Forsøget vil blive registreret hos Datatilsynet og online på www.clinicaltrials.gov.
Alle registrerede data og information vil blive opbevaret hos sponsor (originale data), den forsøgsansvarlige, laboratoriet samt på de lokale hæmodialysecentre (kopier) i 10 år. Biologisk materiale (blod og urin) destrueres efter brug, men der opbevares prøver i en biobank til fremtidig forskning, hvis der opnås samtykke fra forsøgspersonen (se samtykkeerklæring).
Lægemiddelstyrelsen og Videnskabsetisk komité vil modtage information indenfor 90 dage efter projektets afslutning, og en komplet afsluttende rapport vil blive sendt indenfor et år efter afslutningen af projektet. Ved afslutning før tid vil der blive givet besked inden 15 dage.
Monitor på lægemiddelforsøget er De offentlige GCP-enheder i Danmark.

Oplysninger om patienten beskyttes efter lov om behandling af personoplysninger og sundhedsloven, og forsøget vil blive anmeldt til Datatilsynet.

Patientens egen læge vil blive informeret om patientens deltagelse i videnskabeligt forsøg i form af et kort brev tilsendt som epikrise, med mindre forsøgspatienten frabeder sig dette.

FORSIKRING

Forsøgspatienter: Iht. Lov om klage- og erstatningsadgang inden for sundhedsvæsenet LOV nr. 547 af 24/06/2005 er patienterne omfattet af patientforsikringsordningen.
Patienterne gøres opmærksom på dette i form af skriftlig underretning om forsøgspersoners rettigheder.
Sponsor-investigator: er omfattet af hospitalets lovpligtige forsikring.

Medicinalproducent: er omfattet af egen produktansvarsforsikring, som dækker ved fejl på forsøgsmedicinen.

ØKONOMI

Undersøgelsen er initieret af projektgruppen og gennemføres ved hjælp af fondsmidler. Projektmedicinen leveres af en medicinalproducent (Sanofi-Aventis), som ikke har været initiativtager til projektet. Sanofi-Aventis donerer et engangsbeløb til delvis dækning af udgifter til projektmedicinen.

Kontrakt mellem sponsor og Sanofi-Aventis samt tillæg i form af oversættelse af klausulerne til dansk er vedlagt i appendiks.

Den undersøgelsesansvarlige er ikke tilknyttet virksomheder eller fonde, som har interesser i dette projekt.

Der ansøges løbende om fondsmidler. På nuværende tidspunkt er der indløbet tilsagn om donation fra: Nyreforeningens Forskningsfond (50.000,- + 100.000,-), Dansk Nefrologisk Selskabs Forskningsfond (25.000,- + 40.000,-)), Klinisk Institut Annuum (50.000,- + 60.000,-), Beckett-fonden (50.000,-), Overlæge Poul M. Christiansen og hustrus fond (10.000,-), Bjørnows Fond (40.000,-), Aase og Ejnar Danielsens Fond (100.000), Sanofi-Aventis (15.000,-), Snedkermester Sophus Jacobsen og hustru Astrid Jacobsens Fond (50.000,-), Direktør Kurt Bønnelycke og Hustru fru Grethe Bønnelyckes Fond (15.000,-) og Udviklings- og uddannelsesfonden ved afd. C, Aarhus Universitetshospital, Skejby (50.000,-), Fausbølls Helsefond (8.000,-), Frimodt-Heinekes Fond (50.000,-), Kirsten Anthonius' Fond (25.000,-) samt Det Frie Forskningsråd (2.609.875,-).

Fondsmidler indsættes på forskningskonti, som administreres af Aarhus Universitet.

Et eventuelt overskydende støttebeløb anvendes til aflønning af forskningsbioanalytiker eller projektsygeplejerske ved det nyremedicinske forskningslaboratorium, Aarhus Universitetshospital, Skejby. Hvis dette ikke er relevant, tilbagebetales beløbene til donerende fonde.

PUBLIKATION

Resultater af undersøgelsen forventes publiceret i engelsksproget medicinsk tidsskrift, uanset om resultaterne er de forventede eller ej. Forfatterrækkefølgen vil være afhængig af bidragene.

REFERENCELISTE

1. Ng, JohnsonHawley. Is it time to revisit residual renal function in haemodialysis? Nephrology (Carlton) 2007; 12: 209-217.

2. Wang, Lai. The importance of residual renal function in dialysis patients. Kidney Int 2006; 69: 1726-1732.

3. Dialysis - Devices and intradialytic complications. Nephrol Dial Transplant 2006; 21 Suppl 4: iv170-iv181.

4. Foley, ParfreySarnak. Clinical epidemiology of cardiovascular disease in chronic renal disease. Am J Kidney Dis 1998; 32: S112-S119.

5. Frimodt-Moller, Nielsen, Kamper et al. Reproducibility of pulse-wave analysis and pulse-wave velocity determination in chronic kidney disease. Nephrol Dial Transplant 2007

6. Oikawa, Ishihara, Maeda et al. Prognostic value of heart rate variability in patients with renal failure on hemodialysis. Int.Jour Cardiol. 2009; 131(3): 370-377.

7. DNS Landsregisterrapport 2007. 2008

8. Li, Chow, Wong et al. Effects of an angiotensin-converting enzyme inhibitor on residual renal function in patients receiving peritoneal dialysis. A randomized, controlled study. Ann Intern Med 2003; 139: 105-112.

9. Suzuki, Kanno, Sugahara et al. Effects of an angiotensin II receptor blocker, valsartan, on residual renal function in patients on CAPD. Am J Kidney Dis 2004; 43: 1056-1064.

10. Irbesartan T½. 2008

11. van Olden, van Acker, Koomen et al. Time course of inulin and creatinine clearance in the interval between two haemodialysis treatments. Nephrol Dial Transplant 1995; 10: 2274-2280.

12. Brater, Harris, Redfern et al. Renal effects of COX-2-selective inhibitors. Am J Nephrol 2001; 21: 1-15.

13. Williams, LindholmSever. Systolic pressure is all that matters. Lancet 2008; 371: 2219-2221.

14. Bang, Christensen, Hansen et al. Diagnostisk blodtryksmåling - på døgnbasis, hjemme og i konsultationen. 2006

15. Han, Won, Yi et al. No impact of hyperkalaemia with renin-angiotensin system blockades in maintenance haemodialysis patients. Nephrol Dial Transplant 2007; 22: 1150-1155.

16. Blacher, Guerin, Pannier et al. Impact of aortic stiffness on survival in end-stage renal disease. Circulation 1999; 99: 2434-2439.

17. Korevaar, Merkus, Jansen et al. Validation of the KDQOL-SF: a dialysis-targeted health measure. Qual Life Res 2002; 11: 437-447.

18. Molsted, Heaf, Prescott et al. Reliability testing of the Danish version of the Kidney Disease Quality of Life Short Form. Scand J Urol Nephrol 2005; 39: 498-502.

19. GMP. 2008

20. Irbesartan produktresume. 2008

21. Grimes, Schulz. An overview of clinical research: the lay of the land. Lancet 2002; 359: 57-61.

22. Mimura, Takenaka, Kanno et al. Comparison of changes in pulse wave velocity in patients on continuous ambulatory peritoneal dialysis and hemodialysis one year after introduction of dialysis therapy. Adv Perit Dial 2005; 21: 139-145.
