# Supplementary material for: Short and Long-Term Effects of the Angiotensin II Receptor Blocker Irbesartan on Intradialytic Central Hemodynamics: A Randomized Double-Blind Placebo-Controlled One-Year Intervention Trial (the SAFIR Study)
Source: PLoS One. 2015 Jun 1;10(6):e0126882. doi: 10.1371/journal.pone.0126882 (PMC4452642; doi:10.1371/journal.pone.0126882)
Supplement: S2 Protocol — (PDF) [file pone.0126882.s006.pdf]

Skema over undersøgelser

	Ekkokardiografi	Døgnurin (eGFR)	Applanationstonometri	HRV	Transonic	EKG	KDQOL-SF	Kt/V	Blodsukkerskema for diabetikere	Kvartalsblodprøver	Månedsblodprøver	P-Kalium	Forskningsblodprøver	Urinprøver
Besøg 0 - inklusion	X	X												
Besøg 0 - opfølgning														
Før opstart (kvartalsbesøg A)		X	X	X	X	X	X	X	X	X			X	X
1 uge (kvartalsbesøg B)		X	X	X	X	X		X		X			X	X
2 uger												X		
1 måned											X			
6 uger												X		
2 måneder											X			
3 måneder (kvartalsbesøg C)		X	X	X	X	X		X	X	X			X	X
4 måneder											X			
5 måneder											X			
6 måneder (kvartalsbesøg D)		X	X	X	X	X	X	X	X	X			X	X
7 måneder											X			
8 måneder											X			
9 måneder (kvartalsbesøg E)		X	X	X	X	X		X	X	X			X	X
10 måneder											X			
11 måneder											X			
11,5 måneder	X		X											
12 måneder (kvartalsbesøg F)		X	X	X	X	X	X	X	X	X			X	X
Afsluttende besøg														
Drop out / eksklusion	X	X	X	X	X	X	X							
