# Supplementary material for: Short and Long-Term Effects of the Angiotensin II Receptor Blocker Irbesartan on Intradialytic Central Hemodynamics: A Randomized Double-Blind Placebo-Controlled One-Year Intervention Trial (the SAFIR Study)
Source: PLoS One. 2015 Jun 1;10(6):e0126882. doi: 10.1371/journal.pone.0126882 (PMC4452642; doi:10.1371/journal.pone.0126882)
Supplement: S3 Protocol — (DOC) [file pone.0126882.s007.doc]

**Title:**

Renal and cardiovascular effects of irbesartan in dialysis patients - a RCT protocol (SAFIR study)

**Abstract:**

Introduction: Cardiovascular (CV) events are a major cause of morbidity and mortality in haemodialysis (HD) patients. Hypertension, increased arterial stiffness and left ventricular (LV) hypertrophy are highly prevalent and are often poorly controlled. Volume overload is an important factor and treatment strategies that preserve residual renal function (RRF), reduce blood pressure (BP), decrease arterial stiffness and LV hypertrophy could improve survival. Angiotensin II receptor blocker (ARB) treatment can prevent CV events in patients with hypertension and heart failure. However, few data exist in patients with chronic renal failure and it is not known whether ARB-treatment improves clinical outcome in HD-patients.

Material and methods: This is a randomized, controlled, double blinded intervention study. 82 HD patients from six Danish HD centres will be treated for 1 year with an ARB (irbesartan) or placebo. Inclusion criteria are urine output >300 mL/day, dialysis vintage <1 year and LV ejection fraction >30%. The primary outcomes are change in RRF, LV hypertrophy, arterial stiffness and intradialytic haemodynamics.

Conclusion: If ARB-treatment improves RRF and intermediate CV endpoints in a group of newly started HD-patients, it might lead to improved survival for this high risk population.

Funding: The trial is investigator-initiated, investigator driven and supported by the Danish Agency for Science, Technology and Innovation and several private foundations.

Trial registration: Clinical Trials ID: NCT00791830

Keywords:

Chronic renal failure, haemodialysis, angiotensin II receptor blocker, glomerular filtration rate, left ventricular hypertrophy, arterial stiffness, pulse wave velocity, cardiac output

**Introduction**

The mortality rate in incident European dialysis patients is 192 per 1000 person-years compared to 12 in the general population. The most common cause of death is cardiovascular (CV) disease, which accounts for 39% [1].

Dialysis patients often have elevated blood pressure due to volume overload. In addition, chronic renal failure leads to increased arterial stiffness which is reported to be a strong independent risk factor for CV-mortality [2]. The mechanisms causing increased arterial stiffness are incompletely understood. However, it is generally accepted that complete loss of renal function markedly accelerates this process, thereby potentiating traditional CV risk factors such as diabetes, hypercholesterolemia, obesity, and smoking. Furthermore, the loss of kidney function leads to reduced removal and increased cytokine generation as well as impaired immune system [3]. Thus, many dialysis patients have low-grade chronic inflammation [4], which is associated with increased risk of atherosclerotic complications [5].

Different treatment strategies to preserve residual renal function (RRF) and counteract inflammation and development of CV disease have been suggested. Among these are agents blocking the renin-angiotensin-aldosterone-system (RAAS).

In peritoneal dialysis (PD) patients, two Asian open-labelled studies have shown that an angiotensin converting enzyme inhibitor (ACE-I) as well as an angiotensin II receptor blocker (ARB) can preserve RRF [6, 7]. Regarding anti-inflammatory properties and protection against CV disease in haemodialysis (HD) patients, the results of ACE-I and ARB treatment are conflicting, both in short-term and longer follow-up studies [8, 9]. Concerning large artery stiffness, data are scarce whereas left ventricular (LV) mass index is more consistently reported to be positively affected by ARB treatment [9, 10].

Further investigations on ARB treatment in HD patients could be helpful to elucidate the potential to preserve RRF as well as to counteract the development of CV disease.

**Hypotheses**

Irbesartan treatment in newly started HD-patients leads to
- a slower decline of RRF
- stabilization or regression of cardiac hypertrophy
- a decrease in arterial stiffness
- an improvement in intradialytic haemodynamics

**Materials and methods**

Design, patient recruitment and randomization
This study is a double-blind multi-center randomized placebo-controlled intervention trial. Patients are recruited from six Danish HD centres. Eligibility criteria are summarized in Table 1. Inclusion began in May 2009. Last patient last visit is expected in December 2012. Screened patients with urine volume > 300 mL/24h and LV ejection fraction > 30% are randomized to placebo or irbesartan (1:1) by the Pharmacy Department at Aarhus University Hospital (PDAUH). Block randomization is applied according to study site and diabetic status.

Study drug

The study medication consists of the ARB irbesartan 150 mg, or matching placebo. Tablets are delivered from Sanofi-Aventis in blister packages to the PDAUH, which labels blisters for all sites. Code lists, drug labelling, package, and distribution are carried out in accordance with Good Manufacturing Practice.

Study drug is prescribed after baseline investigations are performed at visit A. The initial dose is one tablet per day. After two weeks, daily dose is increased to two tablets, equalling 300 mg of irbesartan, which is the highest recommended dose. If side effects are unacceptable, patients are reduced to one tablet daily.

While in the study, patients can not receive other medications influencing RAAS. Patients prescribed ACE-I, renin inhibitors or ARBs at inclusion stop this medication one week before baseline investigations. All other classes of antihypertensive drugs are accepted. The systolic BP target is 140 mm Hg in all patients.

Study set-up

The patients are investigated at baseline and after one week, to elucidate acute effects of irbesartan, and thereafter every three months for one year (visit A-F). Visit A-F are carried out in the morning two days after HD. Visits after 2 weeks, 1 month, 6 weeks, 2 months and then every month are performed to ensure safety and compliance. Measurements are summarized in Table 2.

Residual renal function

Renal function is followed by measuring glomerular filtration rate (GFR) based on the mean of urinary creatinine and urea clearance. Urine is collected for 24 hours before visit A-F. To minimize urea and creatinine post-dialysis rebound effect, blood samples for creatinine and urea analysis are drawn ten minutes after termination of the HD session two days before visit A-F. Creatinine and urea are also measured before HD at the day of visit A-F. Assuming that interdialytic increase in creatinine and urea concentration is linear, clearance is measured as urinary excretion of creatinine and urea related to the plasma concentrations during the same time interval, Figure 1. Clearance is standardized to a body surface area (BSA) of 1.73 m^2^.

Blood pressure

Blood pressure (BP) is measured before dialysis using validated automated oscillometric BP devices. The patient rests in a sitting position for five minutes with legs uncrossed prior to the measurement. The cuff is positioned at heart level on the arm without an arterio-venous (AV) fistula, with cuff-size matching the circumference of the arm. A minimum of two measurements is performed. In case of > 5 mm Hg deviation in either systolic or diastolic BP, more measurements are performed. The average of the last two is used.

Applanation tonometry

The SphygmoCor® (AtCor Medical Sydney, Australia) system is a widely used device for estimation of central aortic BP and pulse wave velocity (PWV) based on applanation tonometry and is validated for use in patients with chronic renal failure [11]. SphygmoCor® applies a transfer function whereby a non-invasive recording of the pulse wave from the radial artery on the non-AV-fistula arm is transformed to approximate the central aortic pulse wave. Brachial BP is used for calibration and operator index should be >80%. PWV is found by sequential 10-20 sec. recordings of pressure waveforms at the carotid artery (CA) and femoral artery (FA). SphygmoCor® uses the R-wave in an ECG to determine the start of the pulse wave. It is imperative to achieve visually acceptable waveforms and equal heart rates at both sites. Length is approximated by subtracting the distance between the suprasternal notch (SN) and CA from the distance between SN and FA.

Electrocardiograms and HRV

Standard ECGs are obtained before dialysis at visit A-F in order to detect arrhythmias and LV hypertrophy using Sokolow-Lyon and Cornell criteria. In patients with sinus rhythm, heart rate variability (HRV) is assessed with the SphygmoCor® HRV-system SCOR-Hx using a five minute measurement with the patient resting in a supine position and two manoeuvres (Valsalva and standing) selected to challenge the autonomic nervous system.

Echocardiography

Echocardiography (EC) is performed with the patient in the left lateral position by an experienced technician/doctor before study entry and after one year just before end of treatment. Raw data is stored digitally in cineloop format defined by the R wave on the corresponding ECG for off-line analyses using EchoPac software (GE Healthcare).
Quantification of cardiac chamber size, heart valve pathology, LV mass and function are done with treatment allocation concealment by one experienced examiner in accordance with current guidelines [12].

Intradialytic haemodynamics

The Transonic® Hemodialysis Monitor HD02/HD03 and clip-on flow/dilution sensors (Transonic Systems, Inc., USA) is validated for access flow and cardiac output (CO) measurements in HD-patients [13]. CO, brachial BP and heart rate measurements are performed in duplicate within the first and the last 30 minutes of a dialysis session. Mean BP, total peripheral resistance (TPR) and stroke volume (SV) are obtained assuming mean BP = diastolic BP + 1/3 x (systolic BP – diastolic BP)
 and CO = SV x heart rate = mean BP/TPR.

Quality of life questionnaire

Quality of life (QoL) is measured at baseline, at 6 months and before 12 months with the validated Kidney Disease Quality of Life – Short Form (KDQoL-SF), which includes dialysis-related questions [14].

Biochemical measurements

Blood samples are drawn from the fistula cannula or the central venous catheter before start of the HD session. Biochemical measurements are summarized in table 3. Blood sampling at visit A-F are drawn after at least 30 minutes of rest in a supine position with the head elevated to 20^o^. Serum and plasma are frozen immediately after centrifuging. Blood and urine from visit A-F are kept in a biobank at -80^o^C for later use.

Sample size considerations and statistical methods

RRF: In a PD-study, the annual decline in RRF was 3 mL/min/1.73m^2^ without ARB [6]. Assuming a RRF decline=4 mL/min/1.73m^2^/year (due to a faster decline in HD), SD=1.7, type I error=0.05, power=0.80, and the minimal relevant difference =1.4 mL/min/1.73m^2^ (reduction in RRF decline=35%), 24 patients per group are needed.

LV mass index: Based on Kanno et al. [15], a reduction in LV mass index of 23 g/m^2^ in the ARB-treated subjects, 8 g/m^2^ in the placebo group and a SD of 19 g/m^2^ in both groups are assumed. With a power=0.85 and a type I error probability=0.05, 22 patients per group are needed.

PWV: To detect a carotid-femoral PWV difference of 10% (ARB vs. placebo) after 1 year, 22 patients per group are needed. The assumptions are that SD=10% in both groups, power=0.90 and a type I error probability=0.05 [10].

However, in expectation of 40% drop out (e.g. transplantation, adverse events) we decided to recruit 80 patients.

Differences in primary endpoints (e.g. RRF, PWV, LV mass index) between treatment groups over time are investigated using an ANOVA with repeated measurements, which allows for missing values and drop out. Two-sample/paired samples t-tests will be used for comparisons between baseline and end of study. Intention to treat analyses will be performed and p<0.05 is considered significant.

Ethics & GCP

The study is conducted in accordance with good clinical practice (GCP) and the ethical standards described in the Helsinki Declaration. The Central Denmark Region Committees on Biomedical Research Ethics, the Danish Medicines Agency, and the Danish Data Protection Agency have approved the study protocol. All sites are monitored by a local independent GCP-Unit. Clinical Trials ID: NCT00791830.

**Discussion**

The optimal BP level in dialysis patients is debated. Some studies indicate that hypertensive HD patients have a better survival compared to HD patients with normal or low blood pressure [16]. On the other hand, a recent meta-analysis reports better survival among HD patients on antihypertensive medications regardless of their BP levels [17]. We expect BP level to influence the main outcome measures investigated in this study, and a predialytic systolic BP=140 mm Hg is the treatment target in all included patients.
Volume expansion is the most important cause of hypertension in the dialysis population. Consequently, preservation of RRF is important because it allows the patient to excrete salt and water thereby diminishing severe fluid overload and high BP. Furthermore, preserved RRF may improve quality of life for the patient due to a more liberal diet and fluid intake.

Concerning CV endpoints, the value of ARB treatment is not completely elucidated in HD patients. Several small studies indicate that RAAS-blockade is beneficial regarding CV events in HD patients [9]. However, fear of elevated potassium and intradialytic hypotension often results in abandoning ARB treatment in patients starting HD. This aspect is thoroughly investigated in our study.

Increased sympathetic activity from the diseased kidneys is another contributing factor causing elevated BP. HRV is one marker of this [18] which may provide more insight into the degree of sympathetic activation and whether this is affected by ARB.

Left ventricular hypertrophy (LVH) is very common in dialysis patients, and it is reported to be a strong CV risk factor. Increased ventricular muscle mass contributes to coronary risk due to increased oxygen demand. It is also associated with increased myocardial fibrosis and decreased capillary density, which probably serves as a substrate for arrhythmia. Arrythmia is more frequent in patients with LVH and a common cause of death in HD patients. Briefly, the pathogenesis of LVH in dialysis patients can be divided into factors causing increased afterload such as hypertension and increased arterial stiffness and factors causing increased preload such as fluid overload, chronic anemia and AV-fistula. In the present study, the influence of ARB treatment on LVH development is investigated.

The central aortic BP has in several studies been reported to predict CV morbidity and mortality above brachial BP [19]. In addition, antihypertensive drugs seem to have differential effects on central BP despite similar reductions in brachial BP [20]. ARB is known to lower the central BP in non-uremic patients, whereas this study investigates the effect in HD patients.

PWV reflects the stiffness of the aorta and is considered to be a strong predictor for all-cause as well as CV mortality in patients with chronic renal failure [2]. In this study, PWV is measured predialytic six times and once on a non-HD day just before termination of the study period. The aim is to clarify the influence of ARB on PWV progression in HD patients.

Moreover, effects of ARB treatment on biochemical markers reflecting inflammation, RAAS and LV function are systematically investigated.

With our study, we wish to elucidate the effects of ARB on RRF and intermediate CV end points as well as side effects in HD patients. The overall aim is to reduce morbidity and thereby hopefully also mortality in this high-risk patient population.

**References**

1. de Jager DJ, Grootendorst DC, Jager KJ et al. Cardiovascular and noncardiovascular mortality among patients starting dialysis. JAMA 2009;302:1782-9.

2. Blacher J, Guerin AP, Pannier B et al. Impact of aortic stiffness on survival in end-stage renal disease. Circulation 1999;99:2434-9.

3. Kato S, Chmielewski M, Honda H et al. Aspects of immune dysfunction in end-stage renal disease. Clin J Am Soc Nephrol 2008;3:1526-33.

4. Stenvinkel P, Heimbürger O, Paultre F et al. Strong association between malnutrition, inflammation, and atherosclerosis in chronic renal failure. Kidney Int 1999;55:1899-911.

5. Zimmermann J, Herrlinger S, Pruy A et al. Inflammation enhances cardiovascular risk and mortality in hemodialysis patients. Kidney Int 1999;55:648-58.

6. Li PK, Chow KM, Wong TY et al. Effects of an angiotensin-converting enzyme inhibitor on residual renal function in patients receiving peritoneal dialysis. A randomized, controlled study. Ann Intern Med 2003;139:105-12.

7. Suzuki H, Kanno Y, Sugahara S et al. Effects of an angiotensin II receptor blocker, valsartan, on residual renal function in patients on CAPD. Am J Kidney Dis 2004;43:1056-64.

8. Gamboa JL, Pretorius M, Todd-Tzanetos DR et al. Comparative effects of angiotensin-converting enzyme inhibition and angiotensin-receptor blockade on inflammation during hemodialysis. J Am Soc Nephrol 2012;23:334-42.

9. Tai DJ, Lim TW, James MT et al. Cardiovascular effects of angiotensin converting enzyme inhibition or angiotensin receptor blockade in hemodialysis: a meta-analysis. Clin J Am Soc Nephrol 2010;5:623-30.

10. Ichihara A, Hayashi M, Kaneshiro Y et al. Low doses of losartan and trandolapril improve arterial stiffness in hemodialysis patients. Am J Kidney Dis 2005;45:866-74.

11. Frimodt-Moller M, Nielsen AH, Kamper AL et al. Reproducibility of pulse-wave analysis and pulse-wave velocity determination in chronic kidney disease. Nephrol Dial Transplant 2008;23:594-600.

12. Lang RM, Bierig M, Devereux RB et al. Recommendations for chamber quantification: a report from the American Society of Echocardiography's Guidelines and Standards Committee and the Chamber Quantification Writing Group, developed in conjunction with the European Association of Echocardiography, a branch of the European Society of Cardiology. J Am Soc Echocardiogr 2005;18:1440-63.

13. Krivitski NM, Depner TA. Cardiac output and central blood volume during hemodialysis: methodology. Adv Ren Replace Ther 1999;6:225-32.

14. Molsted S, Heaf J, Prescott L et al. Reliability testing of the Danish version of the Kidney Disease Quality of Life Short Form. Scand J Urol Nephrol 2005;39:498-502.

15. Kanno Y, Kaneko K, Kaneko M et al. Angiotensin receptor antagonist regresses left ventricular hypertrophy associated with diabetic nephropathy in dialysis patients. J Cardiovasc Pharmacol 2004;43:380-6.

16. Myers OB, Adams C, Rohrscheib MR et al. Age, race, diabetes, blood pressure, and mortality among hemodialysis patients. J Am Soc Nephrol 2010;21:1970-8.

17. Heerspink HJ, Ninomiya T, Zoungas S et al. Effect of lowering blood pressure on cardiovascular events and mortality in patients on dialysis: a systematic review and meta-analysis of randomised controlled trials. Lancet 2009;373:1009-15.

18. Ranpuria R, Hall M, Chan CT et al. Heart rate variability (HRV) in kidney failure: measurement and consequences of reduced HRV. Nephrol Dial Transplant 2008;23:444-9.

19. Safar ME, Blacher J, Pannier B et al. Central pulse pressure and mortality in end-stage renal disease. Hypertension 2002;39:735-8.

20. Protogerou AD, Stergiou GS, Vlachopoulos C et al. The effect of antihypertensive drugs on central blood pressure beyond peripheral blood pressure. Part II: Evidence for specific class-effects of antihypertensive drugs on pressure amplification. Curr Pharm Des 2009;15:272-89.

**Tables**

Table 1

| Eligibility criteria |
| --- |
| Inclusion criteria |
| 1. Haemodialysis patient |
| 1. Haemodialysis treatment for maximum 12 months |
| 1. Aged 18 years or older |
| 1. Urine volume > 300 mL / 24 hours |
| 1. Contraception if fertile woman |
| 1. Informed consent |
| Exclusion criteria |
| 1. Allergy to angiotensin converting enzyme inhibitors, angiotensin II receptor blockers or renin inhibitors |
| 1. Predialytic systolic blood pressure < 110 mm Hg prior to admission |
| 1. Left ventricular ejection fraction < 30% |
| 1. Myocardial infarction or unstable angina pectoris during the last 3 months prior to admission |
| 1. Not able to comprehend the aims of the project and follow instructions |
| 1. Pregnancy |

Table 2

Summary of study visits and measurements

|  | Screening | 2+6 weeks | Monthly | Visit pre-F | Visit A-F |
| --- | --- | --- | --- | --- | --- |
| Blood pressure | x | x | x | x | x |
| Heart rate, body weight |  | x | x | x | x |
| Blood sampling |  | x (K^+^ only) | x |  | x |
| Urine volume (24 h), GFR | x |  |  |  | X |
| Echocardiography | x |  |  | x |  |
| PWA + PWV |  |  |  | x | x |
| HRV |  |  |  |  | x |
| Cardiac output |  |  |  |  | x |
| ECG |  |  |  |  | x |
| KDQoL-SF (visit A+D+F) |  |  |  |  | x |
| Biobank (blood and urine) |  |  |  |  | x |
| Adverse events registration |  | x | x |  | x |
| Compliance registration |  | x | x |  | x |

Visit A-F: visit A=baseline, visit B=1 week, visit C=3 months, visit D=6 months, visit E=9 months, visit F=12 months). Visit pre-F is performed the day after a haemodialysis session between 11 months and visit F. GFR: Glomerular filtration rate, PWA: pulse wave analysis, PWV: pulse wave velocity, HRV: heart rate variability, ECG: electrocardiogram, KDQoL-SF: quality of life questionnaire.

Table 3

Biochemical measurements

| Blood samples |  | Urine samples |
| --- | --- | --- |
| Monthly and visit A-F | Visit A-F | Visit A-F |
| Albumin | Adrenaline | Albumin |
| Calcium | Aldosterone | Creatinine |
| CO_2_/HCO_3_^-^ | Angiotensin II | Potassium |
| Creatinine | CRP | Sodium |
| Phosphate | Glucose | Urea |
| Potassium | Haemoglobin A_1c_ |  |
| Sodium | Interleukins^1^ |  |
| Urea | Lipids^2^ |  |
|  | Liver function tests^3^ |  |
|  | Noradrenaline |  |
|  | NT-proBNP |  |
|  | Renin |  |
|  | TGF-β |  |

CRP = C-reactive protein, NT-proBNP = N-terminal prohormone of brain natriuretic peptide and TGF-β = Transforming growth factor beta.
1) Interleukins = interleukins (IL-1β, IL-6, IL-8, IL-18).
2) Lipids = total cholesterol, low-density lipoprotein (LDL), high-density lipoprotein (HDL) and triglyceride.
3) Liver function tests = alanine transaminase (ALAT), aspartate transaminase (ASAT), alkaline phosphatise (ALP) and bilirubin.

**Figure legends**

Figure 1
Sampling of blood and urine in relation to haemodialysis sessions

Footnote: HD=haemodialysis.

**Photo legends**

Photo 1

Increased arterial stiffness is common in patients with chronic renal failure and is frequently found even in young patients as illustrated by this conventional X-ray radiography of the right acetabulofemoral joint from a 30 year old female haemodialysis patient with type 1 diabetes showing pronounced arterial calcification of the right femoral artery. Carotid-femoral pulse wave velocity (cfPWV) in this patient was markedly increased (cfPWV≈12 m/s) matching the radiographic findings. The image was obtained because a hip fracture was suspected and calcification was an incidental finding.
