# Supplementary material for: Short and Long-Term Effects of the Angiotensin II Receptor Blocker Irbesartan on Intradialytic Central Hemodynamics: A Randomized Double-Blind Placebo-Controlled One-Year Intervention Trial (the SAFIR Study)
Source: PLoS One. 2015 Jun 1;10(6):e0126882. doi: 10.1371/journal.pone.0126882 (PMC4452642; doi:10.1371/journal.pone.0126882)
Supplement: S1 Table — (DOCX) [file pone.0126882.s008.docx]

**Table S1**

Multivariate repeated measurements model (xtmixed) results

|  | **Test for parallel curves** | **Test for equal levels** | **Test for constant level** |
| --- | --- | --- | --- |
| Parameter | *P* | *P* | *P* |
| ***BP and dialysis parameters*** |  |  |  |
| PreHD systolic BP | 0.05 | 0.4 | **0.005** |
| PreHD diastolic BP | 0.5 | 0.3 | **0.009** |
| PreHD heart rate | 0.1 | 0.7 | 0.5 |
| PreHD weight | 0.08 | 0.4 | 0.7 |
| Ultrafiltration | 0.1 | 0.3 | 0.06 |
| Urine output | 0.2 | 0.3 | **<0.001** |
| PostHD systolic BP | 0.2 | 0.7 | 0.4 |
| PostHD diastolic BP | 0.5 | 0.9 | 0.2 |
| PostHD heart rate | **0.03** | **0.05^a^** | 0.2^b^ |
| Δ(PostHD-PreHD) systolic BP | 0.6† | **0.04^c^** | **0.03^d^** |
| Δ(PostHD-PreHD) diastolic BP | 0.4† | **0.05^c^** | 0.1^d^ |
| ***Blood samples*** |  |  |  |
| Hemoglobin | 0.3 | 0.5 | **0.001** |
| log(Angiotensin II) | **<0.001** | **<0.001^a^** | **<0.001^b^** |
| ***HD_START_*** |  |  |  |
| Cardiac output | 0.9 | 0.4 | 0.5 |
| Systolic BP | 0.1 | 0.5 | 0.1 |
| Diastolic BP | 0.3 | 0.6 | 0.1 |
| Mean blood pressure | 0.3 | 0.6 | 0.2 |
| Heart rate | 0.6 | 0.4 | 0.2 |
| Total peripheral resistance | 0.9 | 0.8 | 0.9 |
| Central blood volume | 0.9 | 0.07 | **0.008** |
| Stroke volume | 0.9 | 0.2 | 0.3 |
| ***HD_END_*** |  |  |  |
| Cardiac output | 0.8 | 0.5 | 0.2 |
| Mean blood pressure | 0.8 | 0.8 | 0.5 |
| Systolic BP | 0.8 | 0.6 | 0.7 |
| Diastolic BP | 0.6 | 0.7 | 0.5 |
| Heart rate | 0.8 | 0.9 | 0.3 |
| Total peripheral resistance | 0.5 | 0.2 | 0.2 |
| Central blood volume | 0.8 | 0.3 | **0.005** |
| Stroke volume | 0.8 | 0.3 | 0.07 |
| ***Δ(HD_END_-HD_START_)*** |  |  |  |
| ΔCardiac output | 0.6 | 0.9 | 0.4 |
| ΔSystolic BP | 0.5 | 0.1 | 0.4 |
| ΔDiastolic BP | **0.05** | **0.02^a^** | 0.06^b^ |
| ΔMean blood pressure | 0.09 | 0.1 | 0.1 |
| ΔHeart rate | 0.8† | 0.04 | 0.2 |
| ΔTotal peripheral resistance | 0.2 | 0.2 | 0.7 |
| ΔCentral blood volume | 0.4 | 0.2 | 0.3 |
| ΔStroke volume | 0.7 | 0.3 | 0.3 |

BP: Blood pressure; HD_START_: Measurements performed within the first 30 minutes of the HD session; HD_END_: Measurements performed within the last 30 minutes of the HD session; a) Model 1 (different development over time) vs. Model 3 (equal levels); b) Model 1 (different development over time) vs. Model 4 (no change over time); c) Model 2 (parallel curves) vs. Model 3 (equal levels): d) Model 2 (parallel curves) vs. Model 4 (no change over time); †) Parallel curves was assumed (Model 2). Mean differences between placebo and ARB with 95% confidence interval were 6.2(0.5-11)mmHg; *P*=0.03 (Δ(PostHD-PreHD)Systolic BP), 3.0(0.1-6.0)mmHg; *P*=0.04 (Δ(PostHD-PreHD)Diastolic BP), and 2.4(0.2-4.6) bpm (*P* = 0.03) (∆Heart rate).
