# Supplementary material for: Short and Long-Term Effects of the Angiotensin II Receptor Blocker Irbesartan on Intradialytic Central Hemodynamics: A Randomized Double-Blind Placebo-Controlled One-Year Intervention Trial (the SAFIR Study)
Source: PLoS One. 2015 Jun 1;10(6):e0126882. doi: 10.1371/journal.pone.0126882 (PMC4452642; doi:10.1371/journal.pone.0126882)
Supplement: S1 Text — (DOCX) [file pone.0126882.s009.docx]

**S1 Text Secondary findings**

Factors influencing cardiac output

CO obtained at HD_START_ correlated significantly with age (*r*=-0.51; *P*<0.001) and plasma hemoglobin level (*r*=-0.31, *P*=0.01) when combining the groups and using all baseline measurements. Mean access flow in the study period (averaged from all available measurements regardless of group) correlated significantly with mean CO at HD_START_ (*r*=0.42; *P* =0.005). Use of CO obtained at HD_END_ showed similar results.

Ultrafiltration volume and correlations with intradialytic changes

UF volume correlated significantly with all intradialytic changes, except for ΔHR, when combining the groups and using all measurements between baseline and twelve months. Accordingly, UF volume was negatively associated with ΔCO (*r*=-0.49; *P*<0.001), ΔSV (*r*=-0.46; *P*<0.001), ΔMAP (*r*=-0.16; *P*=0.003), and ΔCBV (*r* =-0.30; *P*<0.001) and positively associated with ΔTPR (*r*=0.28; *P*<0.001) and ΔHR (*r*=0.10; *P*=0.08). Finally, ΔCO correlated significantly with age (*r*=0.28; *P*<0.001), but there was no significant correlation with access flow (*r*=0.14; *P* =0.09) or plasma hemoglobin level (*r*=-0.06, *P* =0.3). Correlations were similar if only baseline data were used.
